# Smart Polymeric Nanoparticles in Cancer Immunotherapy

**DOI:** 10.3390/pharmaceutics15030775

**Published:** 2023-02-26

**Authors:** Zhecheng Yu, Xingyue Shen, Han Yu, Haohong Tu, Chuda Chittasupho, Yunqi Zhao

**Affiliations:** 1 Wenzhou Municipal Key Lab of Applied Biomedical and Biopharmaceutical Informatics, Wenzhou-Kean University, Wenzhou 325060, China; 2 Zhejiang Bioinformatics International Science and Technology Cooperation Center, Wenzhou-Kean University, Wenzhou 325060, China; 3 Department of Biology, College of Science and Technology, Wenzhou-Kean University, Wenzhou 325060, China; 4 Faculty of Pharmacy, Chiang Mai University, Chiang Mai 50200, Thailand

**Keywords:** cancer, polymer, polymeric nanoparticle, stimuli-responsive, immunotherapy

## Abstract

Cancer develops with unexpected mutations and causes death in many patients. Among the different cancer treatment strategies, immunotherapy is promising with the benefits of high specificity and accuracy, as well as modulating immune responses. Nanomaterials can be used to formulate drug delivery carriers for targeted cancer therapy. Polymeric nanoparticles used in the clinic are biocompatible and have excellent stability. They have the potential to improve therapeutic effects while significantly reducing off-target toxicity. This review classifies smart drug delivery systems based on their components. Synthetic smart polymers used in the pharmaceutical industry, including enzyme-responsive, pH-responsive, and redox-responsive polymers, are discussed. Natural polymers derived from plants, animals, microbes, and marine organisms can also be used to construct stimuli-responsive delivery systems with excellent biocompatibility, low toxicity, and biodegradability. The applications of smart or stimuli-responsive polymers in cancer immunotherapies are discussed in this systemic review. We summarize different delivery strategies and mechanisms that can be used in cancer immunotherapy and give examples of each case.

## 1. Introduction

The global burden of cancer is one of the leading causes of death among all diseases. In 2020, the number of cancer cases was 19.3 million, and the number of cancer-related deaths was 9.96 million [1]. By 2040, it is estimated that there will be 29.5 million new cases of cancer and 16.4 million deaths caused by cancer [2]. In the past 130 years, there have been several significant advances in cancer treatment: radiotherapy, chemotherapy, targeted therapy, and immunotherapy [3,4,5]. Among them, immunotherapy has revolutionized the field of cancer treatment. It overcomes the tumor immune escape mechanism and reawakens the immune system to remove cancer cells. Several types of cancer immunotherapy have been developed, such as chimeric antigen receptor (CAR) T-cell therapy, immune checkpoint inhibitors, monoclonal antibodies, treatment vaccines, and immune system modulators [6]. A number of molecular biomarkers have been selected as key factors for cancer immunotherapy, including programmed death ligand 1 (PD-L1), tumor mutation load (TMB), high microsatellite instability (MSI), and gene mutations [7].

Cancer immunotherapy faces a number of challenges, such as limited patient response, low specificity, immune-related adverse events, and immunosuppression of the tumor microenvironment [8]. Many patients do not benefit from cancer immunotherapy due to its limited effectiveness. Combination therapies have been used to overcome the poor efficacy of cancer immunotherapies. According to preclinical and clinical studies, combination therapies could increase the therapeutic efficacy of cancer treatment but may also cause immune-related adverse effects and lead to higher treatment costs. Meanwhile, many systemically administered immune checkpoint inhibitors have limited tumor specificity, resulting in adverse reactions in normal tissues. The off-target adverse reactions can range from mild symptoms (such as skin redness or blistering) to severe conditions (such as pneumonia, colitis, and endocrinopathies).

Targeted drug delivery aims to deliver therapeutic agents to the action site [9]. Due to their versatility and adaptability to effectively various biological properties, nanoparticles can be used to safely and effectively deliver chemotherapeutic agents. Various nanocarriers have been studied, including polymer-based, lipid-based, carbon-based, and metal-based nanoparticles [10]. Polymers are one of the most commonly used nanomaterials. The polymers used in drug delivery systems should be biocompatible, non-toxic, and free of impurities. In addition, they need to possess a long circulation half-life and a suitable physical structure to accommodate a wide range of active molecules, including small molecules, oligonucleotides, and peptides [11]. Targeted polymer nanoparticles also permit better control of the pharmacokinetics, biodistribution, and bioavailability of drugs. Numerous polymer-based medicines are approved by the FDA or are in clinical trials (Table 1), which will profoundly affect cancer treatment.

Controlled drug delivery is hindered by the process of opsonization. It has been shown that opsonin proteins in blood serum bind rapidly to conventional nanoparticles. These drug delivery carriers are easily recognized and removed by the macrophages of the mononuclear phagocytic system (MPS) before they reach the target site [20]. Therefore, stealth nanoparticles are developed by modifying the surface properties of the carriers with polymers that inhibit opsonin interaction and phagocyte clearance. Various polymers, including natural and synthetic polymers, have been utilized for such purposes. The most commonly used natural polysaccharides are dextran, polysialic acid, hyaluronic acid, chitosan, and heparin. Polyvinyl pyrrolidone, polyacrylamide, polyvinyl alcohol, poly(ethylene glycol) (PEG), and PEG-containing copolymers such as poloxamines, poloxamers, and polysorbates are preferred synthetic polymers [21].

Smart, or stimuli-responsive, polymers may be made from a synthetic or natural resource. It has been demonstrated that polymeric nanoparticles have the advantages of high stability and mass production [22]. Natural polymers used in the pharmaceutical industry include dextrose, chitosan, gelatin, collagen, heparin, and albumin [23]. On the other hand, synthetic polymers such as polyethylene glycol, polylactic acid, poly(lactic-co-glycolic acid), and poly(caprolactone) can also be used in drug delivery [24]. Birrenbach and Speiser pioneered the field and formulated the first nanoparticles for pharmaceutical applications in the early 1970s [25]. Over the past decade, various types of polymers have been evaluated for use in targeted cancer therapy, including polymeric nanoparticles, dendrimers, polymorphs, conjugates, hybrid systems, polymer bodies, and micelles [26]. These nanocarriers can enhance anti-neoplastic activity with minimal side effects while continuously releasing loaded drugs from themself to their target sites [27]. Furthermore, polymeric nanoparticle systems can improve the stability and specificity of the loaded drugs by preventing them from being rapidly metabolized and excreted by the reticuloendothelial system, kidneys, and liver [28,29]. In addition, the drug delivery system with targeted ligand modifications could reduce non-specific distribution and prolong blood circulation [30]. 

Most polymeric nanoparticles are poorly immunogenic or not immunogenic due to their small size. However, some polymeric nanoparticles possess adjuvant properties, such as inducing humoral and cellular immunogenicity [31]. They have the potential to modulate immune responses that can be used in cancer immunotherapy [32]. In addition, the absorption of polymeric nanoparticles by antigen-presenting cells has been shown to induce an effective immune response. They can be taken up by dendritic cells and used for both systemic and mucosal immunotherapy [33].

Polymeric carriers are beneficial for enhancing immunotherapeutic approaches because of their ability to be modified with various physical properties, encapsulants, and surface ligands. Meanwhile, polymeric nanoparticles can be designed to co-deliver multiple therapeutic agents to cancer or immune cells. Research has shown that fine-tuning the size, shape, surface charge, and hydrophobicity of polymeric nanoparticles can improve the delivery of immunotherapeutic agents to tumor tissues [34]. In addition to achieving precise synergistic immunotherapy, polymeric carriers loaded with immunomodulators prevent tumor recurrence and enable long-term immune memory [35]. This approach improves patient response rates while decreasing toxicities and immune-related adverse events.

Smart stimulus-responsive nanoparticles have been extensively studied as efficient vehicles for drug delivery in cancer immunotherapy [36]. Compared to conventional nanomedicines, stimulus-responsive nanoparticles can improve the immune response rate of patients by modulating the tumor microenvironment and converting immune-excluded tumors into immune-inflamed tumors [37]. This review summarizes recent advances in developing a novel stimuli-responsive polymeric drug delivery system that can significantly improve cancer immunotherapy efficacy.

## 2. Synthetic Polymers

**Figure 1:**
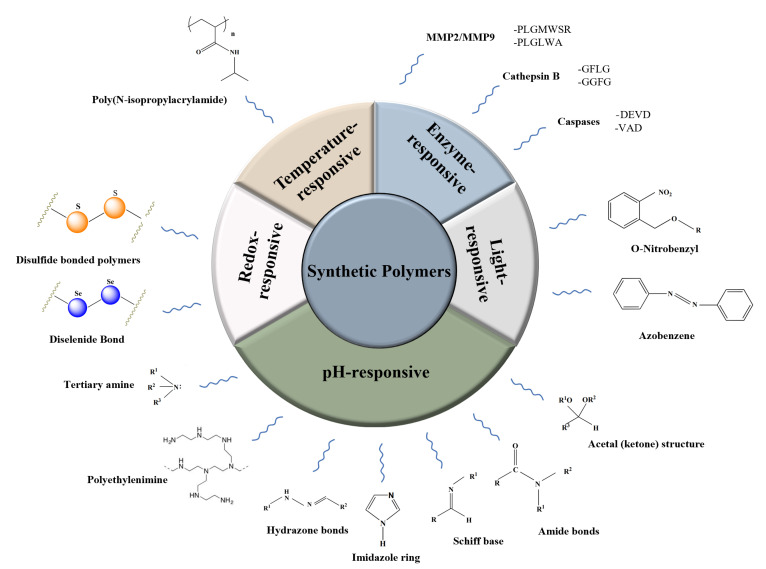
Schematic representation of the stimuli-responsive synthetic polymers.

Synthetic polymers are versatile materials produced by a variety of chemical reactions. Due to their wide range of mechanical, thermal, and degradation properties, synthetic polymers have received increasing attention from the pharmaceutical industry. Over the past several years, synthetic polymer-based smart materials have become one of the most attractive research areas [38] (Figure 1).

### 2.1. Enzyme-Responsive Polymers 

Compared with normal tissues, certain enzymes such as matrix metalloproteinase (MMP), cathepsin B, and caspases are highly expressed in tumor tissues to promote cancer cell growth, invasion, or metastasis [39]. Abnormally expressed enzymes are an essential basis for tumor staging and a material basis for development. Since enzymes are more specific for substrate selection and the reaction conditions are milder, enzyme-based controlled release systems are less likely to cause drug leakage and damage the drug structure during drug delivery [40].

#### 2.1.1. MMP

The extracellular matrix (ECM) contains collagen, elastin, proteoglycan, fibronectin, and other macromolecules [41]. The normal ratio of these extracellular macromolecules is important for the containment of cellular carcinogenesis. MMP is a class of highly expressed proteins found in human cancer cells [42]. There are more than 20 enzymes in the MMP family. In normal physiological processes, MMPs participate in the breakdown of ECM through collagen IV and laminin [43]. MMPs allow tumor cells to enter, invade surrounding tissues, and metastasize in the tumor microenvironment [44]. It has been found that MMP-2 and MMP-9 are highly expressed in a variety of tumor cells, including those in gastric, breast, prostate, rectal, lung, and ovarian cancers. Therefore, MMP-2 and MMP-9 are often selected as targets for enhancing the homing accuracy of nanoparticles and improving efficacy [45]. A peptide chimera (PEG-MP9-aPDL1) was constructed with an MMP-2 cleavable linker for colorectal cancer treatment. Its programmed death-ligand 1 (PD-L1) targeting specificity and favorable pharmacokinetic characteristics made it one of the most promising systemic delivery vehicles. The MP9 used in this study is an optimized stapled oncolytic peptide. It can be released from the conjugation system via MMP-2 cleavage in the tumor microenvironment. Effective tumor lysis and sequestration of immune checkpoints were achieved through this strategy [46].

#### 2.1.2. Cathepsin B 

Cathepsin B, an enzyme in the cysteine protease family, participates in tumor invasion and metastasis [47]. Many tumors express three to nine times more cathepsin B than normal tissues [48]. The tetrapeptide, GFLG, is a cathepsin B-responsive peptide and is stable in the blood. It can be cleaved by cells that secrete high levels of cathepsin [49]. Yang et al. introduced GFLG to the main chain of the N-(2-hydroxypropyl) methacrylamide (HPMA) copolymer–doxorubicin (DOX) conjugate. Cathepsin B cleaved the backbone of the copolymer-drug carriers, releasing the loaded drug from the GFLG peptide chains. The polymer fragments were metabolized by the body and excreted [50]. Using a fluorescent probe (CyA-P-CyB) activated by cathepsin B, Chen et al. designed an effective cancer phototherapy device. Cathepsin B cleaved the linker between CyA-P-CyB in cancer cells, which they fluoresced in the near-infrared region via the Förster resonance energy transfer mechanism. CyA-P-CyB was discovered to have specific phototoxicity towards tumor cells, dependent on cathepsin B activity [51].

A docetaxel-loaded cathepsin B-responsive nanoconjugate was constructed for cancer chemo-immunotherapy. The conjugate was cleaved by cathepsin B in the lysosome to release its payload. The delivery system triggered anti-cancer immune responses by modulating cytotoxic T-cell responses and dendritic cell activity. In a B16 tumor model, synergistic antitumor efficacy was achieved when it was combined with α-PD-1 therapy [52].

#### 2.1.3. Caspases

Cysteine proteases are a set of structurally related cysteine proteases found in cytoplasmic lysates. Aspartate residues are specifically cleaved by caspases, which are essential for apoptosis induction [53]. Different caspases recognize different peptide sequences allowing the development of stimulus-responsive therapeutics.

It is estimated that DEVD is found in less than 1% of all caspase-3/-7 cleavage sites, but remains the optimal recognition sequence for these enzymes [54]. A DEVD-based molecular imaging probe was developed to assess retinal ganglion cell apoptosis through effector caspase activity [55]. In addition, a chimeric peptide, PpIX-1MT, was synthesized by Song et al. The peptide combined the photosensitive PpIX with the immune checkpoint inhibitor 1MT via a DEVD linker to maximize the synergistic effects. The conjugates formed uniformly sized micelles through self-assembly and were then enriched in tumor cells. The PpIX-1MT micelles produced reactive oxygen species upon 630 nm light irradiation, causing cancer cells to apostatize and facilitating the production of tumor antigens capable of triggering an intense immune response. After hydrolyzing the enzyme-sensitive DEVD tetrapeptide, the resulting apoptotic enzyme released the immune checkpoint inhibitor 1-methyltryptophan, which then activated a response from the immune system in vivo to inhibit and clear in situ as well as distant metastatic tumors. Combined with immunotherapy, this approach effectively solves the problem of tumor recurrence and metastasis [56].

### 2.2. pH-Responsive Polymers

The tumor microenvironment has a distinctly acidic character compared to normal tissue. Dysregulated glycolysis in cancer cells results in high lactate levels and an acidic pH in tumor tissues. The pH of tumor tissues typically ranges from 6.5 to 7.2, whereas the pH of intracellular fluid is 5.0 to 6.5, and the pH of lysosomes is 4.5 to 5.0 [57]. Through the process of endocytosis, nanoparticles enter cells and undergo the formation of endosomes. After nanoparticles are fused with lysosomes, the pH values are changed from 6.5 to 4.5 [58].

Since the tumor microenvironment’s pH was lower than that of blood and healthy tissues, extensive research into the direction of pH was initiated [59,60]. The pH-responsive polymer carrier can improve drug delivery efficiency in vivo and reduce adverse drug reactions by utilizing the characteristics of the polymer [61,62]. In recent years, the research on loading drugs into nanoscale carriers began with in-depth research on cancer treatment. Under a normal physiological environment, the loaded drug molecules can be released from the polymer carrier with zero-order kinetic [63]. The released drug molecules could be enriched in tumor tissues to achieve the therapeutic effect. In some pH-sensitive polymers, the proton sponge effect can increase osmotic pressure and disrupt the endosome membrane. Therefore, pH-sensitive carriers in biopharmaceuticals could facilitate endosomal escape and prevent the lysosomal degradation of nucleic acids or proteins [64].

The pH-responsive bonds used in polymer synthesis nanocarriers include Schiff base structures, hydrazone bonds, amido bonds, protonated structures of tertiary amines, and acetal (ketone) structures. [65]. A variety of pH-sensitive polymeric nanoparticles have been formulated, such as microcapsules [66], polymeric vesicles [67], and micelles [68]. Drug molecules were conjugated to the polymer carrier through pH-sensitive chemical bonds, which deformed the configuration of the carrier through the fracture of dynamic chemical bonds, thereby allowing drug release within the target area to be controlled [69].

#### 2.2.1. Schiff Bases 

Schiff bases are compounds containing a C=N group. Under acidic conditions, the structure of Schiff bases is unstable and susceptible to hydrolysis. Acid-sensitive polymeric nano-delivery systems for pH-responsive drug release can be made using this strategy [70].

Imiquimod (R837) can be used in cancer immunotherapy to activate the immune system. To overcome the shortcomings of R837, developing an easy-to-fabricate biocompatible carrier is required. Through acid-responsive Schiff bases, the R837 was conjugated to the amphiphilic biodegradable copolymer methoxy poly(ethylene glycol)-*b*-poly(L-lactide) (mPEG-*b*-PLA). The polymer–drug conjugates were self-assembled into micelles. Under acidic conditions, an accelerated release of R837 from micelles was observed. In RAW264.7 macrophages, R837-loaded micelles stimulate the expression of MHC II-stimulating molecules at pH 6.5, but not at physiologic pH [71].

#### 2.2.2. Polyethyleneimine

A polyethyleneimine (PEI) consists of repeating units of an amine group and a CH_2_CH_2_ spacer [72]. As PEI has good mechanical properties and stability, it has been widely used in developing artificial organs, instruments, and drugs [73]. Therefore, PEI can serve as a co-delivery system for chemotherapy and gene therapy [74].

The nanogels formulated with PDEA-*co*-HP-cyclodextrin-*co*-Pluronic F127 and dimethylmaleic anhydride-modified PEI exhibited excellent drug loading capacity. In tumor microenvironments, loaded chemotherapeutic paclitaxel (PTX) and immunotherapeutic interleukin-2 (IL-2) were rapidly released in response to acidic pH. By improving the maturation of dendritic cells, the nanogel system stimulated anti-tumor immunity, which resulted in the infiltration of immune effector cells in a breast cancer-bearing mouse model [75].

Meng et al. synthesized a polyethyleneimine–lithocholic acid conjugate (2E′). This amphiphilic-modified PEI can self-assemble in aqueous environments. An anionic nucleic acid and small hydrophobic molecules can be co-delivered via the cationic surface and the hydrophobic core of this system. The intrinsic activities of 2E′ components activate antigen-presenting cells. There was a strong anti-tumor response induced by the administration of 2E′ alone or in combination with paclitaxel and siRNA, targeting PD-L1 or cyclic dinucleotide (CDN) in C57BL/6 mice. After a single administration, the treatment significantly enhanced the immunostimulatory effect, resulting in the remission of established tumors and tumor-free survival in the 4T1, CT26, and B16F10 models of tumor growth [76].

Another study developed a PEI-based sequential pH-responsive doxorubicin (DOX) system for combined chemo- and immunotherapy. To make the PEI/CAD complexes more easily adsorbed by polycationic PEI, DOX was converted to pH-responsive cis-aconityl-doxorubicin (CAD). Meanwhile, PEG was modified with aldehydes to shield the PEI/CAD cores from the environment. In the 4T1-bearing mice model, the nanoparticles were successfully coupled with the anti-PD-1 antibody to block the escape of anticancer immunity and maintain the anticancer activity of the T cells activated by immunogenic cell death (ICD) [77].

#### 2.2.3. Imidazole Ring

The imidazole moiety is a five-membered heterocyclic moiety found in many natural products, including histidine, purine, histamine, and DNA [78]. In an acidic environment, the imidazole ring could accept a proton and become hydrophilic [79]. Because imidazole contains two nitrogen atoms, it can form hydrogen bonds, facilitating water solubility. The imidazole moiety is often incorporated into other bioactive frameworks. A major advantage of imidazole-based anticancer drugs is their ability to interfere with DNA synthesis and prevent the growth and division of cancerous cells [80].

To treat breast cancer effectively, a pH-responsive nanoparticle based on the imidazole moiety was developed for a multidimensional chemo-immunotherapeutic modality. Different conformation PLGA polymers were used to load paclitaxel (PTX) and resiquimod (RSQ), an immune activator. In a complex breast cancer plus macrophage cells spheroid model, the pH-responsive star-PLGA nanoparticles showed enhanced drug release and penetration in a pH-dependent manner. In the 4T1-conditioned medium experiment, PTX+RSQ treatment resulted in higher expressions of M1-markers (TNF-α and CD86) than the control group. TNF-α and CD86 levels in nanoparticle-encapsulated drugs were significantly higher than in free drugs. However, although it is an effective chemo-immunotherapy in vitro, further evaluation of the clinical transformation potential of this strategy is required [81].

#### 2.2.4. Hydrazone Bonds

The hydrazone bond is one of the most extensively studied pH-sensitive linkages [82]. The hydrazone bond consists of a hydrazine group (generally the polymer linker) and a carbonyl group (generally the drug linker) cleaved within an acidic tumor microenvironment [83]. Poly(methacrylic acid-co-histidine/doxorubicin/biotin) (HBD) is a pH-sensitive polymer–drug conjugate developed by Wang et al. A hydrazone bond was used to conjugate doxorubicin (DOX) into the polymer. Imiquimod (IMQ), a cancer immunotherapy agent, was also encapsulated to formulate dual cancer-targeting nanoparticles. IMQ and DOX can be released from polymeric nanoparticles in a controllable manner [84].

#### 2.2.5. Tertiary Amine

Polymeric materials containing tertiary amine groups are pH-sensitive, as they can change from negatively charged to positively charged when exposed to hydrogen ions in an acidic environment [85]. In neutral conditions, these polymers can self-assemble into nanoparticles. When the polymers are exposed to a slightly acidic environment at the tumor site, the tertiary amine groups become positively charged and repel each other. As a result of increasing the hydrophilicity of the polymer system, the polymer dissociates and disperses. Thus, the hydrophobic nucleus can release the lipid-soluble drug encapsulated inside and exert its medicinal action [86].

Introducing tertiary amino groups into nanomicelles makes it possible to develop personalized immunotherapy for tumors [87]. This group enabled the design of an acid-responsive nanovaccine that targets the STING pathway. The nanovaccine contains both the antigen and the STING agonist, 5,6-dimethylxantheonone-4-acetic acid (DMXAA). Through intrinsic endosomal escape, nanoparticles promoted the intracytoplasmic release of neoantigens. DMXAA stimulated the STING pathway in dendritic cells, inducing type I interferon secretion and stimulating subsequent T-cell activation. Additionally, tumor-specific T-cell immunity was triggered by neoantigens [88].

#### 2.2.6. Amide Bonds

An amide bond is formed when the carboxylic acid group is substituted with an amino or hydrocarbon amino group. Under acidic conditions, the amide bond can be cleaved, which is one of the most commonly used methods of developing pH-responsive formulations [89].

A pH-mediated endo-stimuli-responsive nanoparticle (en-srNP) can be used for controlled drug release and synergistic immunotherapy against cancer. To improve cellular uptake and extend circulation time, an acid-cleavable PEGylated poly(2-(diethylamino) ethyl methacrylate)@PD-L1-targeting siRNA (PCPP@MTPP@siPDL1) micelle was synthesized. In addition to disassembling the carriers, the micelle core could be rapidly protonated by lysosomes (pH 4.5–6.0), resulting in faster drug release due to disassembly. The formulation demonstrated that the endo-stimuli-responsive nanoparticle promoted the penetration and endocytosis of cancer cells. Immune resistance has been reduced by introducing immune checkpoint inhibitors and activating T lymphocytes that infiltrate tumors [35].

#### 2.2.7. Acetal (Ketone) Structure

As a result of their sensitivity to hydrolysis with a decrease in pH, acetates (including ketals) are promising acid-sensitive linkages [90]. Aldehydes and alcohols are converted into acetals during the condensing process. As a result of hydrolysis under acidic conditions, the acetal can be converted into aldehydes and alcohol [91].

The core of degradable immune-stimulatory nanogels was conjugated with the toll-like receptor (TLR) 7/8 agonist, which is a potent activator of the innate immune system. 2,2-bis(aminoethoxy)propane was used to cross-link the core. The added pH-sensitive ketal moieties enabled acid hydrolysis of the cross-links. The nanogels were excreted from the body in a chain-like polymer state after anti-tumor effects were exerted at the tumor site, avoiding long-term accumulation. As demonstrated by mechanistic studies, the nanogels could diffuse passively into the lymphatic drainage system. An immunological study on mice showed that this formulation induced superior antibodies and robust T-cell responses [92].

### 2.3. Redox-Responsive Polymers

The tumor microenvironment encompasses tumor cells’ structure, function, and metabolic microenvironment and their intrinsic microenvironment [93]. Several factors contribute to the tumor microenvironment (TME), including interstitial fluid pressure, oxygen content, pH value, enzymes, and redox substances within and outside tumor cells. The complexity and specificity of the TME play an important role in cancer occurrence, progression, and metastasis. It has been demonstrated that redox-sensitive nanocarriers can deliver anticancer drugs efficiently and with low adverse effects by taking advantage of the redox gradient between tumor and normal cells [94].

Several reactive oxygen species (ROS) are found in tumor cells, including hydroxyl radicals (·OH), singlet oxygen (^1^O_2_), and hydrogen peroxide (H_2_O_2_). To reduce the damage caused by ROS, tumor cells produce large quantities of reduced substances, such as glutathione (GSH) [95].

In cancer cells, GSH concentrations are approximately 1000 times higher than extracellular GSH and several times higher than normal cells [96]. Therefore, tumor cells are heterogeneous due to abnormally high levels of GSH and ROS. By developing new drug delivery systems, redox-sensitive polymers could deliver drugs to specific cancer cells [97].

#### 2.3.1. Disulfide Bonded Polymers 

Many drug delivery systems have utilized disulfide bonds, including polymeric micelles, nanoparticles, and nanogels [98]. In cancer cells, disulfide bonds are reduced to thiols by GSH, which is highly expressed. They are then oxidized into strongly hydrophilic sulfoxide or sulfone by ROS, thereby increasing the hydrophilicity of the system and prompting drug release [99].

As potential intracellular delivery systems, nanohydrogels with redox-responsive properties have been extensively studied [100]. As hydrophilic nanoparticles, nanohydrogels combine the benefits of both hydrogels and nanoparticles. The advantages of nanohydrogels over solid polymer nanoparticles are their inherent biocompatibility, hydrophilicity, tissue-like mechanical properties, and high porosity. Due to their unique properties, nanohydrogels are particularly suited for encapsulating macromolecular biotherapeutics to facilitate cancer immunotherapy. They are also suitable for systemic drug delivery due to their nanoscale size and hydrophilicity [101]. Disulfide cross-linking schemes have been integrated into nanosized hydrogels to deliver drugs to target sites that contain high levels of reducing agents, such as glutathione (GSH), on demand [102]. 

Nanohydrogels with redox-responsive properties have been demonstrated to be effective in protecting payloads during blood circulation, especially for the release of payloads at the target site, including protein drugs, gene therapeutics, and small-molecule anticancer drugs [103]. Therefore, nanohydrogels with redox-responsive properties are gaining interest as potential therapeutic delivery systems for enhancing the effectiveness of cancer treatment. Antigenic peptides delivered intracellularly to antigen-presenting cells (dendritic cells or macrophages) improve the efficacy of peptide and protein-based cancer immunotherapy [104].

The PEG_2k_-Fmoc-NLG nanocarrier was prepared by conjugating PEG with NLG919, an immune checkpoint inhibitor. The NLG919 and PTX were delivered together using this drug delivery carrier. Compared to the control group, tumors treated with PTX/PEG_2k_-Fmoc-NLG had a higher percentage of CD8^+^ T cells that produced granzyme B. Synergistically, PTX’s tumoricidal properties combined with NLG’s immune-enhancing properties produced much greater anti-tumor effects [105]. 

A similar approach was used in the co-delivery of immunochemotherapy with another anticancer agent. PSSN10 is an immunostimulatory polymeric prodrug carrier with redox-responsive properties. The formulation was designed to deliver NLG919 and doxorubicin (DOX). NLG919 was conjugated to the polymer backbone via a disulfide bond to facilitate the release of the drug from the polymer carrier at the targeted site. In mouse tumor tissues from the PSSN10 treatment group, there was an increase in functional T cells (CD4^+^ and CD8^+^) and fewer Treg cells and MDSCs than in the control group [106].

#### 2.3.2. Diselenide Bond

The redox responsiveness of disulfide has inspired a growing interest in selenium, which, like sulfur, also belongs to the chalcogens. In addition to its physicochemical properties similar to sulfur, selenium plays a crucial role in antioxidant defense and cellular redox homeostasis [107].

Diselenide bonds are reduction-sensitive and can be cleaved by oxidation reactions. Reactive oxygen species are also expressed in high concentrations in tumor cells to bring them into equilibrium with GSH within the cells.

When diselenide bonds are introduced into polymeric drug delivery systems, nanoparticles can exhibit a high degree of redox sensitivity and release drugs with increased precision and completeness. Additionally, prodrug nanoassemblies containing diselenium bonds are more sensitive to H_2_O_2_-triggered drug release than disulfide bonds [108].

A diselenide-pemetrexed assembly combining radio-, chemo-, and natural killer cell-based immunotherapy was developed. Hydrogen bonds were formed between pemetrexed and cytosine-containing diselenides (Cyt-SeSe-Cyt) within the assemblies. As a result of low-dose gamma radiation (5 Gy), commonly used in clinical chemotherapy, selenic acid was formed by the transformation of diselenides, and the assemblies released the loaded pemetrexed. As part of its cancer immunoactivity, selenite inhibited the human leukocyte antigen E (HLA-E) protein on the membranes of cancer cells to activate natural killer (NK) cell anticancer immunity. Furthermore, pemetrexed may increase NK cell IFN-γ production, allowing for the combination of chemotherapy and immunotherapy for cancer treatment [109]. An in vivo study showed that combining immunotherapy, radiotherapy, and chemotherapy using selenium-containing nanoparticles could alleviate the progression of lung metastases and enhance the infiltration of natural killer cells into the tumor microenvironment [110].

### 2.4. Temperature-Responsive Polymeric Delivery Systems

Temperature-responsive drug delivery strategies exploit the local temperature increase that occurs at the focal site to achieve targeted drug release. The temperature of the intra-tumor environment is higher than that of healthy tissue [111]. The subtle change in temperature in the tumor microenvironment is ideal for facilitating thermo-responsive drug release. Thermal-responsive biomaterials have the unique property of changing their solvation state according to temperature. Temperature-responsive release relies on a dramatic change in the physical properties of the thermosensitive material, which triggers drug release in response to local temperature changes around the carrier. The temperature range for drug delivery system activation is generally between 37 and 42 °C, since temperatures above this range may cause protein denaturation [112].

A pentablock copolymer thermal-sensitive hydrogel composed of PEG-PCL-PLA-PCL-PEG with varying ratios of PCL and PLA was developed as a single sustained-release vaccine. A vaccine-encapsulated PLGA nanoparticle or soluble vaccine components were loaded into the hydrogels. This approach increased the complex viscosity of the gel, reduced gel temperature, and minimized the burst release of antigen and adjuvant. A sustained immune response lasting up to 49 days was achieved. In a B16.OVA melanoma mouse model, an antigen-specific immune response was induced by pentablock copolymer hydrogels [113].

**Figure 2:**
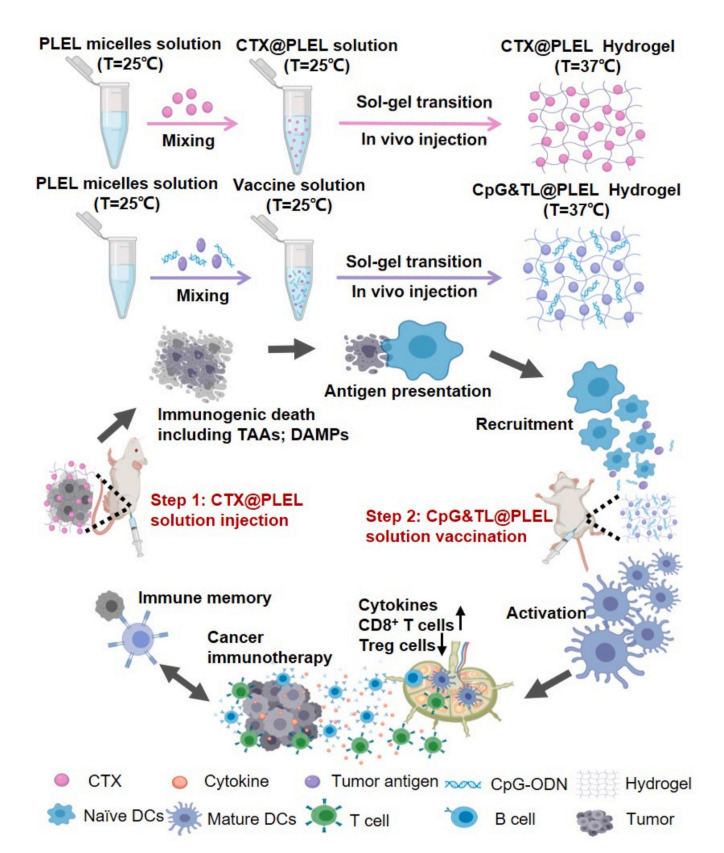
Schematic illustration of the PLEL-based combination strategy to amplify cancer immunotherapy. Adapted with permission from [114], published by Bioactive Materials, 2021.

The development of cancer vaccines based on neoantigens holds great promise in cancer immunotherapy due to their ability to induce an effective antitumor immunity that lasts for a long time. Cyclophosphamide (CTX), an immunogenic cell death (ICD)-inducing drug, is a promising antigen source for cancer vaccines. A CTX-loaded, temperature-responsive poly (D, l-lactide)-poly (ethylene glycol)-poly (D, l-lactide) (PDLLA-PEG-PDLLA, PLEL) hydrogel was designed for CpG combined immunotherapy (Figure 2). In the CT26-bearing mice model, this strategy resulted in a cytotoxic T-lymphocyte response, which inhibits tumor growth and increases survival. By inducing a durable immune memory response, the hydrogel prevented tumor recurrence and distant tumor growth, facilitating the development of commercial tumor vaccines and combining chemotherapy with cancer vaccines in the clinic [114].

Tsai et al. constructed a thermal-responsive hydrogel containing imiquimod (IMQ), an agonist of the toll-like receptors, loaded liposome system for breast cancer immunotherapy. IMQ was first loaded into 1,2-dipalmitoyl-sn-glycero-3-phosphocholine (DPPC) phospholipid liposomes and mixed with the amphiphilic temperature-sensitive hydrogel porous F127. Due to the temperature-sensitive nature of the hydrogel carrier, it can be used as a locoregional therapeutic strategy and reduce the off-target effect. In the 4T1 breast cancer mouse model, a better locoregional therapeutic effect was observed with the thermal-responsive hydrogel carrier compared to traditional intravenous injections, presenting an alternative method for cancer immunotherapy [115].

### 2.5. Light-Responsive Polymeric Nanoparticles

Due to its noninvasive nature and high selectivity, light-responsive therapy is gaining increasing attention in clinical practice. The treatment is safer than traditional treatments such as chemotherapy, surgery, and radiotherapy [116]. Near-infrared (NIR) light with wavelengths ranging from 750 to 2000 nm was found to be more effective in locating deeper positions in tissue [117]. NIR photoimmunotherapy (PIT) uses an antibody–photosensitizer conjugate and NIR to achieve cancer immunotherapy with selective cell death in targeted tumor cells, low toxicity to normal cells in the surrounding environment, and activation of an anti-tumor immune response [118,119]. This strategy could kill tumor cells in the targeted position under the excitation of a photosensitizer [120]. For example, some components exert photodynamic therapy (PDT) effects, such as IR-780, which could convert oxygen into cytotoxic reactive oxygen species (ROS) [121]. Some other components present photothermal therapy (PTT) effects, such as reduced graphene oxide–iron oxide nanoparticles applied to produce local heating or general hyperthermia [122]. In addition, under the damage signals caused by this strategy, NIR-PIT will release tumor-specific antigens to induce local dendritic cell activation, which in turn activates T cells, leading to T cell proliferation and its mediated tumor cell killing, which is also called immunogenic cell death (ICD) [119].

**Figure 3:**
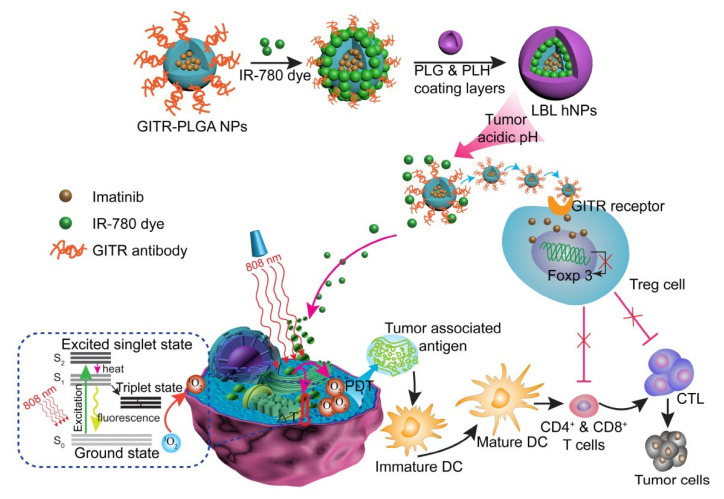
Schematic illustration of NIR therapy and regulatory T cell modulation using layer-by-layer hybrid nanoparticles. Adapted with permission from [123], published by Theranostics, 2018.

Ou et al. developed a pH-sensitive layer-by-layer hybrid nanoparticle (glucocorticoid-induced TNF receptor family-related protein/poly(lactic-co-glycolic acid, GITR-PLGA) loaded with IR-780 and imatinib (IMT) for cancer photoimmunotherapy (Figure 3). To prevent degradation and achieve stimulus-responsive effects in targeted locations under NIR exposure, the GITR-PLGA core was coated with a poly-L-histidine (PLH) and poly(ethylene glycol)-block-poly(L-glutamic acid) (PEG-b-PLG) layer. In B16/BL6 and MC-38 tumor-bearing mouse models, this system successfully eradicated tumor growth by apoptosis induction and tumor-associated antigen production. Meanwhile, CD8+ T cells were activated, and the suppressive function of Treg cells was reduced [123].

A multifunctional phase-transformation nanoparticle was designed for melanoma immunotherapy and was evaluated in the B16F10-luc mouse model. The formulation encapsulated anti-PD1 antibody (aPD1), iron oxide, and perfluoropentane (PFP) in a PLGA shell modified with polyethylene glycol (PEG) and Gly-Arg-Gly-Asp-Ser (GRGDS) peptides. This strategy enhanced the delivery efficacy of aPD1. Moreover, under the laser irradiation, the iron oxide further activated dendritic cells to drive the tumor-specific effector T cell response and induce tumor cell death and the production of tumor-associated antigens, which consequently increased CD8^+^ T cell infiltration in the tumor site [124].

## 3. Natural Polymers

**Figure 4:**
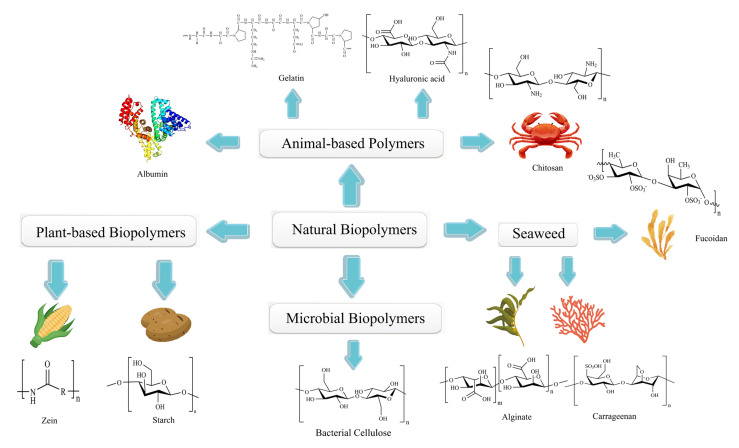
Schematic representation of the natural biopolymers.

Naturally occurring biopolymers can be found in plants, animals, microbes, and marine organisms [125] (Figure 4). They are often used in biomedicine and the food industry. For example, food-grade colloids can be produced from animal proteins and polysaccharides, and gelatin polymers can be used for wound dressings [126]. It has been demonstrated that natural biopolymers have several beneficial properties over synthetic polymers, including bioadhesion, multiple bioactivities, excellent biocompatibility, low toxicity, and biodegradability [127]. Furthermore, most natural biopolymers are biocompatible, ensuring their distinct properties. Currently, most innovative natural products and drugs approved by the US Food and Drug Administration (FDA) are derived from microorganisms and plants. Crofelemer, for example, is derived from the red latex of the *Peruvian croton*, a plant in the *Euphorbiaceae* family. This biopolymer is the first oral botanical prescription drug approved by the FDA for treating non-infectious diarrhea symptoms in HIV/AIDS patients receiving antiretroviral therapy [128].

Natural biopolymers are great candidates for drug delivery carriers in targeted cancer therapy. As with synthetic polymers, naturally occurring polymers can be modified to respond to a variety of stimuli. Meanwhile, some natural biopolymers can modulate the immune system or be used as immunoadjuvants. However, the use of natural polymers in constructing smart polymeric nanoparticles for cancer immunotherapy still needs further investigation. This section reviews the natural polymers that have the potential to be designed as stimuli-responsive nanocarriers for cancer immunotherapeutic strategies.

### 3.1. Animal-Based Biopolymers

#### 3.1.1. Albumin

Albumin is a small globular protein synthesized by the liver. In blood circulation, albumin has crucial clinical significance and important physiological functions in living organisms, including maintaining stable plasma colloid osmotic pressure, ensuring the exchange between intracellular fluid, extracellular fluid, and tissue fluid, and playing a colloidal protective and stabilizing role for globulins [129]. Albumin is a non-specific transporter that forms soluble complexes by reversibly binding with many small, insoluble organic molecules in the body [129]. It could also automatically bind to heavy metal ions in the body and act as a detoxification agent [130]. In addition, albumin is crucial for regulating the plasma pH and maintaining colloidal osmotic pressure. It plays a key role in improving bioavailability and ensuring the proper transport of long-chain fatty acids, nutrients, ions, and various medicines administered systemically [131]. As a natural polymer, albumin is biocompatible, biodegradable, non-toxic, and low immunogenic. It has been used as a drug delivery vehicle for the topical administration of vulnerable organs, such as the eyes [132].

NanoPcM, a pH-sensitive photothermal nanomaterial, was constructed through the self-assembly of morpholine-modified silicone phthalocyanine (PcM) and serum albumin for cancer-targeted photodynamic immunotherapy. Since serum albumin showed a strong interaction with PcM, it was used to prevent severe PcM aggregation in the aqueous solution. The fabricated NanoPcM could induce Type I ROS generation and endow a significantly enhanced immunogenic photodynamic therapeutic (PDT) effect against hypoxic cancer cells. The synergistic use of NanoPcM-based PDT in combination with αPD-1-based immunotherapy also significantly inhibited 4T1 cancer cell growth, diminished spontaneous lung metastasis, and triggered tumor regression in the BALB/c mouse model. This strategy induced an abscopal effect by modulating the tumor immune microenvironment and enhancing immune responses throughout the body [133].

In another study, PTX was loaded into human serum albumin nanoparticles through albumin-binding technology. An acid-sensitive linker was used to conjugate the monoclonal anti-PD-L1 antibody to the PTX-loaded serum nanoparticle. The PD-L1-targeted albumin nanoparticle loaded with PTX (PD-L1/PTX@HSA) was used for combinational chemo-immune therapy and showed enhanced drug release at low pH levels in cancer cells. Combining PD-L1/PTX@HSA with cytotoxic T-lymphocyte antigen-4, immune responses were accentuated due to the synergetic effect. Infiltration of effector T-cells, immunosuppressive PD-L1, and regulatory T-cell suppression was successfully observed [134].

#### 3.1.2. Gelatin

Gelatin, a macromolecular hydrocolloid, can be produced by irreversible animal collagen acid or base hydrolysis [135]. Due to its biocompatibility and biodegradability, gelatin has been used as a multifunctional ingredient in the pharmaceutical industry and medicine [136]. The swelling effect of gelatin makes it a valuable raw material for plasma substitutes, and gelatin hydrogel has been used as an injectable biomaterial in surgery [137].

Gelatin and Pluronic^®^ F127, an FDA-approved polymer, formed an innovative thermo-responsive hydrogel based on biomaterials. This co-polymer hydrogel platform was used for GSNO (a NO-donor) and cytotoxic T-lymphocyte-associated protein-4 (CTLA-4) monoclonal antibody (mAb) locoregional delivery in a controlled manner. The unique properties of this hydrogel system enabled the tumor microenvironment to trigger drug release and micelle formation for lymphatic uptake. The systemic co-delivery of antagonizing CTLA-4 mAb and GSNO using the hydrogel system resulted in synergistic anticancer effects in a monotherapy-resistant melanoma model. The synergistic effect of the two interventions was also observed in the 4T1 breast cancer-bearing mice model. This hydrogel system improved cancer immunotherapy’s local and abscopal effects by facilitating the modulation of immunological pathways relevant to anticancer immune response regulation [138].

Bu et al. developed a self-assembling gelatinase-sensitive nanoparticle (Gel-N-ICG) comprising indocyanine green (ICG), a photosensitizer, and NSC74859, a signal transducer activator of transcription 3 (STAT3) inhibitor. By combining photothermal therapy with multifunctional immunotherapy, Gel-N-ICG inhibited the growth of head and neck squamous cell carcinomas (HNSCCs). After the Gel-N-ICG was degraded by the tumor microenvironment’s overexpressed gelatinase, MMP-2/9, the loaded ICG and NSC were released. In addition to the effective photothermal destruction of tumors by ICG, NSC74859 induced potent anti-tumor immunity that enhances the effectiveness of cancer treatment. Gel-N-ICG NPs could inhibit tumor microenvironment immunosuppression and improve anticancer efficacy in the Tgfbr1/Pten 2cKO mouse HNSCC model. Compared to the control group, the nanoparticle significantly reduced CD11b^+^ Gr1^+^ myeloid-derived suppressor cells (MDSCs) in the spleen, blood, and tumor tissue. Meanwhile, the population of PD-1 also decreased after the Gel-N-ICG nanoparticle treatment [139].

#### 3.1.3. Hyaluronic Acid

Hyaluronic acid (HA) is one of the most abundant polysaccharides in the human body. This polymer is composed of D-glucuronic acid and N-acetyl-D-glucosamine [140]. Due to its non-toxic, biodegradable, and cytocompatible properties, HA has been used in a variety of drug delivery platforms, such as micelles, hydrogels, and polymer–drug conjugates [141]. CD44 is one of the binding receptors for HA. By interacting with HA, the CD44 receptor mediates interactions between cells and between cells and the matrix. Adhesion to the HA molecule plays an important role in cancer cell proliferation, differentiation, and migration. Therefore, it has been suggested that the interaction between CD44 and HA may be a potential therapeutic target for cancer treatment [142,143].

An HA-based hydrogel system was synthesized for cancer chemo-photothermal immunotherapy. Polydopamine (PDA), as a cross-liner, was conjugated to the thiolated HA, conferring excellent photothermal properties to the hydrogel. Doxorubicin (DOX) and CpG-ODN, an immune adjuvant, were loaded into the HA-hydrogel system. The unique properties of this hydrogel system enable the accelerated release of DOX into surrounding tumor tissue upon irradiation with near-infrared light. The DOX/CpG-ODN-loaded HA/PDA-Gel showed a synergistic anti-proliferation effect against breast cancer. Using the hydrogel system, the high photothermal effect kills cancer cells and releases chemotherapeutic drugs into tumor tissue. Cancer-associated antigens were generated, and a pan-semiconductor-specific immune response was induced. The tumor tissue apoptotic cell number in the chemophotothermal group (DOX@PDA+) and three-modality combination group (CpG@DOX@PDA+) was higher than that of single-modality therapy. Meanwhile, the HA-hydrogel formulation inhibited distant metastatic cancer cell proliferation by improving the systemic immune response. In CpG@DOX@PDA(+) and CpG@PDA(+) groups, TNF-α and IL-6 contents in serum and CD3^+^/CD8^+^ T cells levels in the spleen were higher than in other treatment groups. This study showed that a strong immune response was induced by photothermal therapy plus an immune adjuvant [144].

Wang et al. established an HA-based macrophage-mediated delivery system (M@C-HA/ICG) that possessed the characteristic of M1-subtype macrophage phenotypes for photo/immune therapy. Macrophage tropism was used to improve nanomedicine’s accumulation and retention time in tumors. To formulate the M@C-HA/ICG, the thermal-conversion-based carbon nanoparticle was modified with HA, and the photosensitizer indocyanine green (ICG) was loaded into the delivery system. The drug delivery system activated macrophages to release pro-inflammatory factors. After laser irradiation, M@C-HA/ICG increased TNF-α and IL-2 production in the 4T1 tumor-bearing model. Meanwhile, ICG triggered type II photooxidation to generate reactive oxygen species (ROS). M@C-HA/ICG showed outstanding anticancer efficacy by enhancing tumor accumulation. Moreover, cytomembrane damage caused by ROS, apoptosis induced by heat, and pro-inflammatory cytokine-mediated immunotherapy were observed [145].

#### 3.1.4. Chitosan

Chitin is the second most abundant polysaccharide, and chitosan is its deacetylated form [146]. Chitosan is composed of randomly distributed deacetylated units, β-(1-4)-linked D-glucosamine, and acetylated units, acetyl-D-glucosamine [147]. It is a non-toxic, biodegradable, and biocompatible polymer approved by the US FDA and assured safe [148]. Chitosan has been widely used in medicine and pharmacy, including tissue engineering, drug delivery, ophthalmology, wound healing, and vaccine adjuvants [149].

Chitosan can regulate the immune system by activating natural killer cells, increasing macrophage accumulation and activation, inducing interferon and interleukin production in mitotic cells, inducing cytokines, and enhancing delayed-type hypersensitivity [150]. Therefore, chitosan can strengthen the immune effects and antibody response to inhibit tumor growth. In gene therapy, chitosan can bind to DNA/RNA because of the electrostatic interactions, protecting it from nuclease degradation. In addition, owing to its excellent physicochemical properties, using chitosan as a carrier can achieve specific targeted delivery and improve the biological activity, stability, and solubility of the therapeutic agents [151].

As a biomimetic vaccine, a pH-responsive chitosan-based polymeric micelle was made for lymph nodes’ targeted delivery to enhance the anticancer immune response. Chitosan/ovalbumin micelles grafted with histidine-modified stearic acid could inhibit tumor growth by promoting T-cell immune response induction and antitumor-related cytokine secretion. This strategy allowed the vaccine to be directly delivered to the lymph nodes and taken up by the dendritic cells. The pH-dependent property of the micelle was used to control antigen release and accumulation at the target sites. The micelle formulation inhibited tumor growth in the C57BL/6 mouse model by inducing robust anti-tumor immune responses, such as higher levels of TNF-α, IL-12, and IL-6 than the control group [152].

### 3.2. Plant-Based Biopolymers

Polysaccharides consist of multiple monosaccharide units linked together by glycosidic bonds [153]. Plant polysaccharides consist primarily of glucose, galactose, fructose, xylose, arabinose, fucose, rhamnose, mannose, and uronic acid [154]. Each of these polymers consists of different types of monosaccharides polymerized in a particular proportion, and they differ in their main compositions. Plant polysaccharides have been used in clinical cancer immunotherapy due to their immunomodulatory, anti-tumor, antibacterial, and antiviral properties [155,156].

#### 3.2.1. Starch

Starch is a polysaccharide of glucose molecules. Starch can be produced by most green plants and is considered one of the major energy resources for living creatures. Due to its low cost, true biodegradability, and renewability, starch has been used in the medical and pharmaceutical industries [157]. As the temperature rises, starch’s swelling and water solubility also increase [158]. It can be used as a tablet disintegrant or a binder. 

Cyclodextrin is an oligosaccharide consisting of a ring of glucose subunits linked by glycosidic bonds. The enzymatic transformation of starch results in the production of cyclodextrin [159]. As well as being used in the food, pharmaceutical, drug delivery, and chemical industries, cyclodextrins are also used in agriculture and the environment [160]. Cyclodextrins have hydrophobic interiors and hydrophilic exteriors. As a drug delivery vehicle, it has been used to encapsulate hydrophobic molecules. Solubility, stability, and bioavailability can be improved using this strategy. In addition, various targeting moieties can be conjugated with cyclodextrin to achieve specific targeting [161].

A cyclodextrin-based supramolecularly assembled programmable immune activating nanodrug (PIAN) was constructed. The programmable nanodrug was assembled supramolecularly through the interactions between poly[(N-2-hydroxyethyl)-asparagine]-Pt(IV)/β-cyclodextrin (PPCD), CpG/polyamido-thioxo-adamantane (CpG/PAMAM-TK-Ad), and methoxy poly(ethylene glycol)-thioxo-adamantane (mPEG-TK-Ad). Following intravenous administration, the complex accumulated at the targeted site. In the tumor microenvironment, elevated ROS promoted PIAN dissociation and the release of the payload. This led to the activation of antigen-presenting cells, antigen presentation, and a robust anticancer immune response. At least two types of cancer were effectively activated using this strategy by stimulating all steps in the immune activation process. This strategy established a novel approach to developing nanomedicine that can be programmed as an immunotherapeutic in situ vaccine for cancer treatment [162].

#### 3.2.2. Zein

Zein is a class of natural prolamine proteins derived from corn [163]. Due to its low cytotoxicity, reproducibility, and high drug-binding ability, zein is often used as a drug delivery carrier and mainly as a film-forming material in the food, pharmaceutical, and chemical industries [164]. Zein has a unique solubility property. It is neither soluble in water nor in anhydrous alcohol. However, it can be dispersed in a 60–95% alcohol–water solution, making it an ideal natural nutritional preservative [165].

The immunogenicity of zein is controversial. Food-grade zein is less immunogenic than collagen. However, zein may induce an immune response in celiac patients via antigen-presenting cells, resulting in specific interactions between zein and IgA. Furthermore, the hydrophilic surface formed by the PEGylation of zein inhibits macrophage uptake and renders the immunogenic response [164].

Zein has been used to fabricate a drug delivery system. Oral zein-based nanoparticles protect therapeutic proteins against digestion in the gut environment. The folate-modified zein framework could also achieve active targeting of macrophages [166]. In addition, a doxorubicin-loaded zein nanoparticle showed that the release profile was governed by various parameters, including the hydrolysis process and the pH value [167]. In acidic conditions, accelerated drug release was also observed in paclitaxel-loaded zein nanoparticles [168].

### 3.3. Microbial Biopolymers

The natural polymers produced by microbes include polysaccharides (bacterial cellulose and dextran), polyamides (poly-glutamic acid), and polyesters (polyhydroxy-alkanoate). A bacterial cellulose injectable suspension immobilized with ^131^I-labeled anti-PD-L1 antibody (αPD-L1) was prepared for cancer radioimmunotherapy to address the problems of low response rates and immune-related adverse events caused by immune checkpoint blockade. The bacterial cellulose nanofibers comprised an intricate network structure that contributed to the long-term retention of ^131^I-labeled αPD-L1 within tumors. The formulation reduced the side effects associated with the non-specific distribution of ^131^I. In addition, immobilized ^131^I-PD-L1 can enhance immunogenic cell death and stimulate the maturation of multiple immune cells that induce a systemic anti-tumor immune response [169].

### 3.4. Biopolymers from Seaweed

A number of marine biopolymers can be harvested at a relatively low cost, such as fucoidan, alginate, and carrageenan, all of which are renewable, stable, and non-toxic [170]. Marine-based biopolymers possess various structural characteristics that make them suitable for a wide range of biomedical applications. It has been demonstrated that marine biopolymer-based drugs possess a variety of biological functions, including anticancer, antibacterial, tissue regeneration, antioxidant, and antiaging properties [171]. The anticancer properties of marine biopolymers deserve special attention. The US FDA has approved many essential cancer medicines derived from marine sources, including cytarabine and ticlopidine [172].

#### 3.4.1. Fucoidan

Fucoidan is referred to as fucoidan sulfate derived from brown algae and echinoderms [173]. It is one of the most important marine drugs currently being developed due to its ability to inhibit the growth of tumor cells and treat cancer [174]. Fucoidan, a known immunomodulator, may be used as an adjuvant in the induction of anticancer immunity in animals and humans [175]. In addition, fucoidan was suggested to act as a mucosal adjuvant to enhance the immunotherapeutic effects of immune checkpoint inhibitors against metastatic lung cancer [176]. 

A fucoidan-based theranostic nanoplatform (FM@VP) was developed to treat triple-negative breast cancer (TNBC), overcome hypoxia, and enhance photodynamic therapy and anti-tumor immunity. The multifunctional nanoparticle cluster was formed by the co-assembly of a fucoidan polymer with a reducible PAMAM dendrimer, verteporfin (VP), and MnO_2_ nanoparticles. In this system, MnO_2_ is an oxygen-evolving nanomaterial that responds to the tumor microenvironment. The glutathione-sensitive nanocomplex demonstrated a number of therapeutic benefits, including synergetic photodynamic and TNBC-targeted therapy, as well as the ability to elicit an immune response. Furthermore, FM@VP alleviated hypoxia in tumors, inhibited oncogenic signaling, and reduced cancer-mediated immunosuppression. Both anti-tumor immunity and anti-metastasis activity were significantly enhanced [177].

#### 3.4.2. Alginate

The polyanionic copolymer alginate (ALG) consists of the sugar residues mannuronic and guluronic, possessing carboxyl groups in the chain [178]. The material is highly biocompatible and biodegradable. Multivalent cation alginate hydrogel has been extensively used in drug delivery and cell encapsulation [179].

Two different types of alginate (ALG) were cross-linked by CaCl_2_ to formulate functionalized alginate nanoparticles. For dendritic cell targeting, mannose-modified ALG (MAN-ALG) was used. A model antigen, ovalbumin (OVA) was conjugated to ALG (ALG=OVA) using a pH-sensitive linker. The MAN-ALG/ALG=OVA nanoparticles increased antigen uptake and cytosolic antigen release in BMDCs. In C57BL/6 mice, subcutaneous administration of this formulation also induced a significant cytotoxic T lymphocyte response and inhibited the E.G7 tumor growth. It suggested that MAN-ALG/ALG=OVA nanoparticles would be an effective cancer immunotherapy nanovaccine [180].

An antigen and adjuvant co-delivery platform (APPC) was developed for liver cancer immunotherapy with good cytocompatibility and histocompatibility. Through electrostatic interactions, polyanionic alginate (ALG) and polycationic polyethyleneimine (PEI) was used to deliver the glypican-3 peptide antigen as well as an unmethylated cytosine-phosphate-guanine (CpG) adjuvant. In addition to promoting antigen uptake by dendritic cells (DCs) and stimulating their maturation, APPC can facilitate antigen delivery into the cytoplasm through escape from endosomes. In Hepa1–6 cells, APPC induced a specific CTL response, which led to an increase in the infiltration of CD4^+^ helper T lymphocytes and spleen cytolytic CD8^+^ T lymphocytes [181].

#### 3.4.3. Carrageenan

The sulfated polysaccharide carrageenan (CGN) is a TLR4 ligand extracted from red algae [182]. Recent studies have demonstrated that CGN is an effective antiviral agent for treating papillomavirus infections [183]. The structure of CGN is similar to that of heparan sulfate, a cell-attachment factor for HPV. Therefore, using carrageenan as an adjuvant may enhance the efficacy of HPV peptide vaccines [184].

There are three general categories of CGNs classified based on the position and number of sulfated esters, including kappa (κ)-, iota (ι)-, and lambda (λ)-CGNs. Through the TLR4 signaling pathway, λ-carrageenan promoted DC maturation and could be used as an adjuvant for DC-based vaccines. In addition, the treatment with λ-carrageenan enhanced the ability of DCs to stimulate allogeneic spleen cell growth. The combination of λ-carrageenan-DC (HPV-CGN-DC) and HPV peptide induced strong CD8^+^ T cell responses and inhibited TC-1 cancer growth in a mouse model. The levels of CD4^+^ and CD8^+^ T cells, as well as their activation status, were significantly increased in the mouse model. In contrast, natural regulatory T cells and CD11b^+^Gr-1^+^ cells levels were significantly decreased [185].

El-Deeb et al. demonstrated that alginate/-carrageenan oral microcapsules containing MH751906 from *Agaricus bisporus* have immunotherapeutic effects against colon cancer by activating NK cells resident in the gut. The microcapsules demonstrated excellent acidic stability, controlled release, and high-temperature stability in a pH-mimicking colonic environment. After activation with microcapsules, NK cell populations of CD16^+^CD56^+^ were significantly increased. It was found that 74.09 percent of the activated NK cells exhibited cytotoxic effects. Most human colon cancer Caco-2 cells were arrested at G_0_/G_1_ phase, and apoptosis was observed [186].

## 4. Conclusions and Perspectives

Smart synthetic polymers used in cancer immunotherapies were discussed in three aspects: enzyme-, pH-, and redox-responsive. In terms of safety and efficacy, smart polymers offer significant advantages over conventional drug delivery systems [187]. This approach can increase the amount of drug in circulation, improve the stability and biocompatibility of the drug in circulation, enhance the targeting of nanoparticles to tumors, and decrease drug leakage in circulation and accumulation in normal tissues [188]. The efficacy of the drug could be improved by enhancing the cellular uptake of target cells [188]. Studies indicate that smart polymeric nanoparticles could improve tumor immunotherapy, relieve immunosuppression, and prevent cancer cells from escaping the immune system [189]. Smart stimulus-responsive synthetic biopolymers may help with cancer immunotherapy.

Although remarkable achievements have been made in smart polymeric nanoparticle development, this strategy still possesses several issues. For example, different tissues have different microenvironments and pHs. The pH-responsive nanoparticles need to ensure that they can overcome these barriers and remain intact before entering cancer cells. In addition, the heterogeneity of tumor cells may make it difficult to achieve controllability based on redox molecular mechanisms. The development of stimulus-responsive polymer drug delivery systems may facilitate tumor immunotherapy [35]. The combination of different stimulus-response strategies can play a mutually beneficial and complementary role in overcoming the weaknesses of a single stimulus response. The integrated strategy has the potential to lead to major advancements in tumor therapy.

Natural biopolymers can be derived from animals, plants, marine organisms, microorganisms, etc. [127]. These biopolymers are biodegradable and biocompatible. These natural biopolymers can be combined with nanotechnology to provide multifunctional nanodrug carriers for cancer immunotherapy [190]. The nanoformulation reduces the cytotoxicity of the payload or coating carrier and prevents the degradation of the drug. The use of biomedical engineering materials is also restricted by the fact that some of them have poor mechanical properties (e.g., alginates), are insoluble in common reagents (e.g., chitosan), or are immunogenic or antigenic (e.g., gelatin) [191].

Currently, biopolymer hydrogels are still in the proof-of-concept phase of cancer immunotherapy. Biopolymers and degradants are being investigated in terms of their long-term biosafety [192]. By adjusting immunotherapy at different stages, immune-specific reporter genes can be used to monitor the effects of immunotherapy [193]. Meanwhile, due to the limitations of near-infrared light, PTT combined with immunotherapy can only treat superficial tumors. The treatment of internal tumors can be combined with other penetrating external interventions. The immune system of mice differs from that of humans. Other animal models may be used to evaluate the therapeutic effect and complete the clinical translation process. To test these possibilities, more investigation in this direction is urgently needed in the future [194].

## Figures and Tables

**Table 1:** Polymeric nanoparticles in clinical use or under clinical trials.

Polymeric Platform	Active Component	Drug Product	Indication	Status	Reference or Identifier
Micelles	Paclitaxel	Genexol-PM	Breast cancer and small cell lung cancer	Marketed in Europe, Republic of Korea	[12]
	Paclitaxel	Apealea	First relapse of platinum-sensitive epithelial ovarian cancer, primary peritoneal cancer, and fallopian tube cancer	Approved by EMA ^1^	[13]
	Paclitaxel	Abraxane	Pancreatic cancer, NSCL and breast cancer	Approved by US FDA	[14]
	Docetaxel	-	Advanced solid tumors	Phase 2 (Not yet recruiting)	NCT05254665
Polymeric Nanoparticles	Leuprolide acetate	Eligard	Prostate cancer	Approved by US FDA ^2^	[15]
	iNKT ^3^ activator (ThrCer6, IMM60) ^4^; NY-ESO-1 ^5^ cancer-testis antigen peptides	PRECIOUS-01 *	Advanced solid tumor	Phase 1 (Recruiting)	NCT04751786
	Tumor lysate ^6^; GM-CSF ^7^; CpG ^8^	WDVAX *	Melanoma	Phase 1(Active, not recruiting)	NCT01753089
Polymer–protein conjugate	Asparaginase	Pegaspargase (Oncaspar)	Acute lymphoblastic leukemia	Approved by US FDA	[16]
	IL-15 receptor agonist	NKTR-255 *	Non-Hodgkin lymphoma; relapsed/refractory diffuse large B-cell lymphoma	Phase 2/3 (Recruiting)	NCT05664217
Polymer–drug conjugate	Paclitaxel	FID-007	Advanced malignant solid neoplasm; refractory malignant solid neoplasm	Phase 1 (Recruiting)	NCT03537690
	Lidocaine	ST-01	Postoperative pain; postsurgical pain	Phase 2 (Recruiting)	NCT05193227
	Rotigotine	SER-214	Parkinson’s disease	Phase 1 (Active, not recruiting)	NCT02579473
	Ammonium molybdate	BP-C2	Prostate cancer	Phase 1 (Not yet recruiting)	NCT04186585
	Quercetin	Nano-QUT ^9^	Oral cancer	Phase 2 (Not yet recruiting)	NCT05456022
	Triptorelin pamoate	Decapeptyl	Prostate cancer	Phase 3 (Recruiting)	NCT05458856
	Mitoxantrone	DHAD-PBCA- NPs	Hepatocellular carcinoma	Phase 2 (Recruiting)	[17]
	Doxorubicin	BA-003	Hepatocellular carcinoma	Phase 3 (Recruiting)	[18]
	Docetaxel	ABI-008	Prostate cancer	Phase 1/2 (Recruiting)	NCT00477529
	DACHPt	ProLindac	Advanced ovarian cancer	Phase 2/3 (Recruiting)	[19]
	Rapamycin	ABI-009	Solid tumors	Phase 1/2 (Completed)	NCT02494570
	Paclitaxel	Nanotax	Peritoneal and neoplasms	Phase 1 (Completed)	NCT00666991

EMA ^1^: European Medicines Agency; US FDA ^2^: US Food and Drug Administration; iNKT ^3^: invariant natural killer T cell; (ThrCer6, IMM60) ^4^: threitolceramide-6; NY-ESO-1 ^5^: New York Esophageal Squamous Cell Carcinoma-1; tumor lysate ^6^: proteins from participants’ melanoma cells; GM-CSF ^7^: granulocyte–macrophage colony-stimulating factor; CpG ^8^: cytosine–phosphate–guanine; Nano-QUT ^9^: quercetin-encapsulated PLGA-PEG nanoparticles; * means the drug applied for cancer immunotherapy.

## Data Availability

Not applicable.

## Abbreviations

The following abbreviations are used in this manuscript:

·OH	Hydroxyl radicals
1MT	1-methyltryptophan
^1^O_2_	Singlet oxygen
αPD-L1	Anti-PD-L1 antibody
ALG	Alginate
CAD	Cis-aconityl-doxorubicin
CDN	Cyclic dinucleotide
CGN	Carrageenan
CpG	Cytosine–phosphate–guanine
CpG8	Cytosine–phosphate–guanine
CTLA-4	Cytotoxic T-lymphocyte-associated protein-4
Cyt-SeSe-Cyt	Cytosine-containing diselenides
DCs	Dendritic cells
DEVD	Asp-Glu-Val-Asp
DMXAA	5,6-dimethylxantheonone-4-acetic acid
DOX	Doxorubicin
ECM	Extracellular matrix
EMA	European Medicines Agency
en-srNP	Endo-stimuli-responsive nanoparticle
FDA	US Food and Drug Administration
GFLG	Gly-Phe-Leu-Gly
GGFG	Gly-Gly-Phe-Gly
GITR	Glucocorticoid-induced TNF receptor
GM-CSF	Granulocyte-macrophage colony-stimulating factor
GRGDS	Gly-Arg-Gly-Asp-Ser
GSH	Glutathione
H_2_O_2_	Hydrogen peroxide
HA	Hyaluronic acid
HLA-E	Human leukocyte antigen E
HNSCCs	Head and neck squamous cell carcinomas
HPMA	N-(2-hydroxypropyl) methacrylamide
ICD	Immunogenic cell death
ICG	Indocyanine green
IL-2	Interleukin-2
IMQ	Imiquimod
IMT	Imatinib
iNKT	Invariant natural killer T cell
LBL hNPs	Layer-by-layer hybrid nanoparticles
mAb	Monoclonal antibody
MAN	Mannose
MDSCs	Myeloid-derived suppressor cells
MMP	Matrix metalloproteinase
mPEG-b-PLA	Methoxy poly(ethylene glycol)-b-poly(L-lactide)
mPEG-TK-Ad	Methoxy poly(ethylene glycol)-thioxo-adamantane
Nano-QUT	Quercetin-encapsulated PLGA-PEG nanoparticles
NK	Natural killer
NY-ESO-1	New York Esophageal Squamous Cell Carcinoma-1
OVA	Ovalbumin
PAMAM-TK-Ad	Polyamido-thioxo-adamantane
PCL	Polycaprolactone
PcM	Morpholine-modified silicone phthalocyanine
PDA	Polydopamine
PD-L1	Programmed death-ligand 1
PDT	Photodynamic therapeutic
PEG	Polyethylene glycol
PEG-b-PLG	Poly(ethylene glycol)-block-poly(L-glutamic acid)
PEG-PCL-PLA-PCL-PEG	Polyethylene glycol -polycaprolactone -poly(propyleneglycol) -poly(caprolactone-poly(ethylene glycol)
PEI	Polyethyleneimine
PFP	Perfluoropentane
PIAN	Programmable immune activating nanodrug
PIT	Photoimmunotherapy
PLA	Polypropylene glycol
PLGA	Poly(lactic-co-glycolic acid)
PLGLWA	Pro-Leu-Gly-Leu-Trp-Ala
PLGMWSR	Pro-Leu-Gly-Met-Trp-Ser-Arg
PLH	Poly-L-histidine
PPCD	Poly[(N-2-hydroxyethyl)-asparagine]-Pt(IV)/β-cyclodextrin
PpIX	Protoporphyrin IX
PTT	Photothermal therapy
PTX	Paclitaxel
ROS	Reactive oxygen species
RSQ	Resiquimod
STAT3	Signal transducer activator of transcription 3
ThrCer6, IMM60	Threitolceramide-6
TLR	Toll-like receptor
TME	Tumor microenvironment
TNBC	Triple-negative breast cancer
VAD	Val-Ala-Asp
VP	Verteporfin

## References

[1] Xia C., Dong X., Li H., Cao M., Sun D., He S., Yang F., Yan X., Zhang S., Li N. (2022). Cancer Statistics in China and United States, 2022: Profiles, Trends, and Determinants. Chin. Med. J. (Engl.).

[2] Bray F., Ferlay J., Soerjomataram I., Siegel R.L., Torre L.A., Jemal A. (2018). Global Cancer Statistics 2018: Globocan Estimates of Incidence and Mortality Worldwide for 36 Cancers in 185 Countries. CA A Cancer J. Clin..

[3] Ackerknecht E.H. (1980). The History of Cancer Therapy. Gesnerus.

[4] DeVita V.T., Chu E. (2008). A History of Cancer Chemotherapy. Cancer Res..

[5] Helmy K.Y., Patel S.A., Nahas G.R., Rameshwar P. (2013). Cancer Immunotherapy: Accomplishments to Date and Future Promise. Ther. Deliv..

[6] Zhang Y., Zhang Z. (2020). The History and Advances in Cancer Immunotherapy: Understanding the Characteristics of Tumor-Infiltrating Immune Cells and Their Therapeutic Implications. Cell. Mol. Immunol..

[7] Wang D.R., Wu X.L., Sun Y.L. (2022). Therapeutic Targets and Biomarkers of Tumor Immunotherapy: Response Versus Non-Response. Signal Transduct. Target. Ther..

[8] Whiteside T.L., Demaria S., Rodriguez-Ruiz M.E., Zarour H.M., Melero I. (2016). Emerging Opportunities and Challenges in Cancer Immunotherapy. Clin. Cancer Res. Off. J. Am. Assoc. Cancer Res..

[9] Moghtaderi M., Sedaghatnia K., Bourbour M., Fatemizadeh M., Salehi Moghaddam Z., Hejabi F., Heidari F., Quazi S., Farasati Far B. (2022). Niosomes: A Novel Targeted Drug Delivery System for Cancer. Med. Oncol. (Northwood Lond. Engl.).

[10] Zhu W., Wei Z., Han C., Weng X. (2021). Nanomaterials as Promising Theranostic Tools in Nanomedicine and Their Applications in Clinical Disease Diagnosis and Treatment. Nanomaterials.

[11] Tewari A.K., Upadhyay S.C., Kumar M., Pathak K., Kaushik D., Verma R., Bhatt S., Massoud E.E.S., Rahman M.H., Cavalu S. (2022). Insights on Development Aspects of Polymeric Nanocarriers: The Translation from Bench to Clinic. Polymers.

[12] Gupta A., Costa A.P., Xu X., Burgess D.J. (2021). Continuous Processing of Paclitaxel Polymeric Micelles. Int. J. Pharm..

[13] Borges G.S.M., Lima F.A., Carneiro G., Goulart G.A.C., Ferreira L.A.M. (2021). All-Trans Retinoic Acid in Anticancer Therapy: How Nanotechnology Can Enhance Its Efficacy and Resolve Its Drawbacks. Expert Opin. Drug Deliv..

[14] Bobo D., Robinson K.J., Islam J., Thurecht K.J., Corrie S.R. (2016). Nanoparticle-Based Medicines: A Review of Fda-Approved Materials and Clinical Trials to Date. Pharm. Res..

[15] Sartor O. (2003). Eligard: Leuprolide Acetate in a Novel Sustained-Release Delivery System. Urology.

[16] Dinndorf P.A., Gootenberg J., Cohen M.H., Keegan P., Pazdur R. (2007). Fda Drug Approval Summary: Pegaspargase (Oncaspar) for the First-Line Treatment of Children with Acute Lymphoblastic Leukemia (All). Oncologist.

[17] Zhou Q., Sun X., Zeng L., Liu J., Zhang Z. (2009). A Randomized Multicenter Phase II Clinical Trial of Mitoxantrone-Loaded Nanoparticles in the Treatment of 108 Patients with Unresected Hepatocellular Carcinoma. Nanomed. Nanotechnol. Biol. Med..

[18] Barraud L., Merle P., Soma E., Lefrançois L., Guerret S., Chevallier M., Dubernet C., Couvreur P., Trépo C., Vitvitski L. (2005). Increase of Doxorubicin Sensitivity by Doxorubicin-Loading into Nanoparticles for Hepatocellular Carcinoma Cells In Vitro and In Vivo. J. Hepatol..

[19] Nowotnik D.P., Cvitkovic E. (2009). Prolindac (Ap5346): A Review of the Development of an HPMA DACH Platinum Polymer Therapeutic. Adv. Drug Deliv. Rev..

[20] Fam S.Y., Chee C.F., Yong C.Y., Ho K.L., Mariatulqabtiah A.R., Tan W.S. (2020). Stealth Coating of Nanoparticles in Drug-Delivery Systems. Nanomaterials.

[21] Bao J., Zhang Q., Duan T., Hu R., Tang J. (2021). The Fate of Nanoparticles in Vivo and the Strategy of Designing Stealth Nanoparticle for Drug Delivery. Curr. Drug Targets.

[22] Zielińska A., Carreiró F., Oliveira A.M., Neves A., Pires B., Venkatesh D.N., Durazzo A., Lucarini M., Eder P., Silva A.M. (2020). Polymeric Nanoparticles: Production, Characterization, Toxicology and Ecotoxicology. Molecules.

[23] Nayak A., Olatunji O., Bhusan Das D., Vladisavljević G., Olatunji O. (2016). Pharmaceutical Applications of Natural Polymers. Natural Polymers: Industry Techniques and Applications.

[24] Sung Y.K., Kim S.W. (2020). Recent Advances in Polymeric Drug Delivery Systems. Biomater. Res..

[25] Kanaani L., Ebrahimi Far M., Kazemi S.M., Choupani E., Mazloumi Tabrizi M., Ebrahimi Shahmabadi H., Akbarzadeh Khiyavi A. (2017). General Characteristics and Cytotoxic Effects of Nano-Poly (Butyl Cyanoacrylate) Containing Carboplatin on Ovarian Cancer Cells. Asian Pac. J. Cancer Prev. APJCP.

[26] Mu W., Chu Q., Liu Y., Zhang N. (2020). A Review on Nano-Based Drug Delivery System for Cancer Chemoimmunotherapy. Nano-Micro Lett..

[27] Vincent M.P., Navidzadeh J.O., Bobbala S., Scott E.A. (2022). Leveraging Self-Assembled Nanobiomaterials for Improved Cancer Immunotherapy. Cancer Cell.

[28] Chan J.M., Valencia P.M., Zhang L., Langer R., Farokhzad O.C. (2010). Polymeric Nanoparticles for Drug Delivery. Methods Mol. Biol. (Clifton N.J.).

[29] Sinha R., Kim G.J., Nie S., Shin D.M. (2006). Nanotechnology in Cancer Therapeutics: Bioconjugated Nanoparticles for Drug Delivery. Mol. Cancer Ther..

[30] Alexis F., Pridgen E., Molnar L.K., Farokhzad O.C. (2008). Factors Affecting the Clearance and Biodistribution of Polymeric Nanoparticles. Mol. Pharm..

[31] Al-Nemrawi N.K., Darweesh R.S., Al-Shriem L.A., Al-Qawasmi F.S., Emran S.O., Khafajah A.S., Abu-Dalo M.A. (2022). Polymeric Nanoparticles for Inhaled Vaccines. Polymers.

[32] Yang Z., Ma Y., Zhao H., Yuan Y., Kim B.Y.S. (2020). Nanotechnology Platforms for Cancer Immunotherapy. Wiley Interdiscip. Rev. Nanomed. Nanobiotechnol..

[33] Jia Y., Omri A., Krishnan L., McCluskie M.J. (2017). Potential Applications of Nanoparticles in Cancer Immunotherapy. Hum. Vaccines Immunother..

[34] Craparo E.F., Bondì M.L. (2012). Application of Polymeric Nanoparticles in Immunotherapy. Curr. Opin. Allergy Clin. Immunol..

[35] Zhang J., Lin Y., Lin Z., Wei Q., Qian J., Ruan R., Jiang X., Hou L., Song J., Ding J. (2022). Stimuli-Responsive Nanoparticles for Controlled Drug Delivery in Synergistic Cancer Immunotherapy. Adv. Sci..

[36] Tong R., Langer R. (2015). Nanomedicines Targeting the Tumor Microenvironment. Cancer J. (Sudbury Mass.).

[37] Shen L., Huang Y., Chen D., Qiu F., Ma C., Jin X., Zhu X., Zhou G., Zhang Z. (2017). pH-Responsive Aerobic Nanoparticles for Effective Photodynamic Therapy. Theranostics.

[38] Koide H. (2021). Design of Synthetic Polymer Nanoparticles That Capture and Neutralize Target Molecules. Yakugaku Zasshi J. Pharm. Soc. Jpn..

[39] Chuanjun Liu C.Z., Zhang X. (2020). Application and Progress of Functional Peptides in Tumor Therapy. Univ. Chem..

[40] Kessenbrock K., Plaks V., Werb Z. (2010). Matrix Metalloproteinases: Regulators of the Tumor Microenvironment. Cell.

[41] Berthier C., Marti H.P. (2006). Metzincins, Including Matrix Metalloproteinases and Meprin, in Kidney Transplantation. Swiss Med. Wkly..

[42] Madsen M.A., Deryugina E.I., Niessen S., Cravatt B.F., Quigley J.P. (2006). Activity-Based Protein Profiling Implicates Urokinase Activation as a Key Step in Human Fibrosarcoma Intravasation. J. Biol. Chem..

[43] Malemud C.J. (2006). Matrix Metalloproteinases (MMPs) in Health and Disease: An Overview. Front. Biosci. A J. Virtual Libr..

[44] Stamenkovic I. (2000). Matrix Metalloproteinases in Tumor Invasion and Metastasis. Semin. Cancer Biol..

[45] Roomi M.W., Monterrey J.C., Kalinovsky T., Rath M., Niedzwiecki A. (2009). Patterns of MMP-2 and MMP-9 Expression in Human Cancer Cell Lines. Oncol. Rep..

[46] Lu L., Zhang H., Zhou Y., Lin J., Gao W., Yang T., Jin J., Zhang L., Nagle D.G., Zhang W. (2022). Polymer Chimera of Stapled Oncolytic Peptide Coupled with Anti-PD-L1 Peptide Boosts Immunotherapy of Colorectal Cancer. Theranostics.

[47] Mort J.S., Buttle D.J. (1997). Cathepsin, B. J. Biochem. Cell Biol..

[48] Aggarwal N., Sloane B.F. (2014). Cathepsin B: Multiple Roles in Cancer. Proteom. Clin. Appl..

[49] Zhang C., Pan D., Li J., Hu J., Bains A., Guys N., Zhu H., Li X., Luo K., Gong Q. (2017). Enzyme-Responsive Peptide Dendrimer-Gemcitabine Conjugate as a Controlled-Release Drug Delivery Vehicle with Enhanced Antitumor Efficacy. Acta Biomater..

[50] Yang Y., Pan D., Luo K., Li L., Gu Z. (2013). Biodegradable and Amphiphilic Block Copolymer-Doxorubicin Conjugate as Polymeric Nanoscale Drug Delivery Vehicle for Breast Cancer Therapy. Biomaterials.

[51] Chen X., Lee D., Yu S., Kim G., Lee S., Cho Y., Jeong H., Nam K.T., Yoon J. (2017). In vivo near-Infrared Imaging and Phototherapy of Tumors Using a Cathepsin B-Activated Fluorescent Probe. Biomaterials.

[52] Du H., Zhao S., Wang Y., Wang Z., Chen B., Yan Y., Yin Q., Liu D., Wan F., Zhang Q. (2020). pH/Cathepsin B Hierarchical-Responsive Nanoconjugates for Enhanced Tumor Penetration and Chemo-Immunotherapy. Adv. Funct. Mater..

[53] Hug H., Los M., Hirt W., Debatin K.M. (1999). Rhodamine 110-Linked Amino Acids and Peptides as Substrates to Measure Caspase Activity Upon Apoptosis Induction in Intact Cells. Biochemistry.

[54] Julien O., Wells J.A. (2017). Caspases and Their Substrates. Cell Death Differ..

[55] Barnett E.M., Zhang X., Maxwell D., Chang Q., Piwnica-Worms D. (2009). Single-Cell Imaging of Retinal Ganglion Cell Apoptosis with a Cell-Penetrating, Activatable Peptide Probe in an in Vivo Glaucoma Model. Proc. Natl. Acad. Sci. USA.

[56] Song W., Kuang J., Li C.X., Zhang M., Zheng D., Zeng X., Liu C., Zhang X.Z. (2018). Enhanced Immunotherapy Based on Photodynamic Therapy for Both Primary and Lung Metastasis Tumor Eradication. ACS Nano.

[57] Chen W., Meng F., Li F., Ji S.J., Zhong Z. (2009). pH-Responsive Biodegradable Micelles Based on Acid-Labile Polycarbonate Hydrophobe: Synthesis and Triggered Drug Release. Biomacromolecules.

[58] Ma X., Wang Y., Zhao T., Li Y., Su L.C., Wang Z., Huang G., Sumer B.D., Gao J. (2014). Ultra-pH-Sensitive Nanoprobe Library with Broad pH Tunability and Fluorescence Emissions. J. Am. Chem. Soc..

[59] Zhang X., Lin Y., Gillies R.J. (2010). Tumor pH and Its Measurement. J. Nucl. Med. Off. Public Soc. Nucl. Med..

[60] Kellum J.A. (2000). Determinants of Blood pH in Health and Disease. Crit. Care.

[61] Deirram N., Zhang C., Kermaniyan S.S., Johnston A.P.R., Such G.K. (2019). pH-Responsive Polymer Nanoparticles for Drug Delivery. Macromol. Rapid Commun..

[62] Pang X., Jiang Y., Xiao Q., Leung A.W., Hua H., Xu C. (2016). pH-Responsive Polymer-Drug Conjugates: Design and Progress. J. Control. Release Off. J. Control. Release Soc..

[63] Xiang J., Zhao R., Wang B., Sun X., Guo X., Tan S., Liu W. (2021). Advanced Nano-Carriers for Anti-Tumor Drug Loading. Front. Oncol..

[64] Varkouhi A.K., Scholte M., Storm G., Haisma H.J. (2011). Endosomal Escape Pathways for Delivery of Biologicals. J. Control. Release Off. J. Control. Release Soc..

[65] Dai S., Ravi P., Tam K.C. (2008). pH-Responsive Polymers: Synthesis, Properties and Applications. Soft Matter.

[66] Chen J., Dong X., Feng T., Lin L., Guo Z., Xia J., Tian H., Chen X. (2015). Charge-Conversional Zwitterionic Copolymer as pH-Sensitive Shielding System for Effective Tumor Treatment. Acta Biomater..

[67] Johnson R.P., Uthaman S., John J.V., Lee H.R., Lee S.J., Park H., Park I.K., Suh H., Kim I. (2015). Poly(Pega)-B-Poly(L-Lysine)-B-Poly(L-Histidine) Hybrid Vesicles for Tumoral pH-Triggered Intracellular Delivery of Doxorubicin Hydrochloride. ACS Appl. Mater. Interfaces.

[68] Liao J., Peng H., Liu C., Li D., Yin Y., Lu B., Zheng H., Wang Q. (2021). Dual pH-Responsive-Charge-Reversal Micelle Platform for Enhanced Anticancer Therapy. Mater. Sci. Eng. C Mater. Biol. Appl..

[69] Liu J., Huang Y., Kumar A., Tan A., Jin S., Mozhi A., Liang X.J. (2014). pH-Sensitive Nano-Systems for Drug Delivery in Cancer Therapy. Biotechnol. Adv..

[70] Wu X., Yu G., Luo C., Maeda A., Zhang N., Sun D., Zhou Z., Puntel A., Palczewski K., Lu Z.R. (2014). Synthesis and Evaluation of a Nanoglobular Dendrimer 5-Aminosalicylic Acid Conjugate with a Hydrolyzable Schiff Base Spacer for Treating Retinal Degeneration. ACS Nano.

[71] Li Z., Gao J., Xiang Z., Zhang H., Wang Y., Zhang X. (2021). A pH-Responsive Polymer Linked with Immunomodulatory Drugs: Synthesis, Characteristics and in Vitro Biocompatibility. J. Appl. Toxicol. JAT.

[72] Kauffman A.C., Piotrowski-Daspit A.S., Nakazawa K.H., Jiang Y., Datye A., Saltzman W.M. (2018). Tunability of Biodegradable Poly(Amine- Co-Ester) Polymers for Customized Nucleic Acid Delivery and Other Biomedical Applications. Biomacromolecules.

[73] Lee H., Lee Y., Statz A.R., Rho J., Park T.G., Messersmith P.B. (2008). Substrate-Independent Layer-by-Layer Assembly by Using Mussel-Adhesive-Inspired Polymers. Adv. Mater..

[74] Fahira A.I., Amalia R., Barliana M.I., Gatera V.A., Abdulah R. (2022). Polyethyleneimine (PEI) as a Polymer-Based Co-Delivery System for Breast Cancer Therapy. Breast Cancer (Dove Med. Press).

[75] Shang L., Jiang X., Yang T., Xu H., Xie Q., Hu M., Yang C., Kong L., Zhang Z. (2022). Enhancing Cancer Chemo-Immunotherapy by Biomimetic Nanogel with Tumor Targeting Capacity and Rapid Drug-Releasing in Tumor Microenvironment. Acta Pharm. Sinica. B.

[76] Meng F., Wang J., He Y., Cresswell G.M., Lanman N.A., Lyle L.T., Ratliff T.L., Yeo Y. (2022). A Single Local Delivery of Paclitaxel and Nucleic Acids Via an Immunoactive Polymer Eliminates Tumors and Induces Antitumor Immunity. Proc. Natl. Acad. Sci. USA.

[77] Jiang M., Chen W., Yu W., Xu Z., Liu X., Jia Q., Guan X., Zhang W. (2021). Sequentially pH-Responsive Drug-Delivery Nanosystem for Tumor Immunogenic Cell Death and Cooperating with Immune Checkpoint Blockade for Efficient Cancer Chemoimmunotherapy. ACS Appl. Mater. Interfaces.

[78] Siwach A., Verma P.K. (2021). Synthesis and Therapeutic Potential of Imidazole Containing Compounds. BMC Chem..

[79] Chen J.X., Wang M., Tian H.H., Chen J.H. (2015). Hyaluronic Acid and Polyethylenimine Self-Assembled Polyion Complexes as pH-Sensitive Drug Carrier for Cancer Therapy. Colloids Surf. B Biointerfaces.

[80] Ali I., Lone M.N., Aboul-Enein H.Y. (2017). Imidazoles as Potential Anticancer Agents. MedChemComm.

[81] Maravajjala K.S., Swetha K.L., Roy A. (2022). pH-Responsive Nanoparticles for Multidimensional Combined Chemo-Immunotherapy of Cancer. J. Pharm. Sci..

[82] Sonawane S.J., Kalhapure R.S., Govender T. (2017). Hydrazone Linkages in pH Responsive Drug Delivery Systems. Eur. J. Pharm. Sci. Off. J. Eur. Fed. Pharm. Sci..

[83] Howard M.D., Ponta A., Eckman A., Jay M., Bae Y. (2011). Polymer Micelles with Hydrazone-Ester Dual Linkers for Tunable Release of Dexamethasone. Pharm. Res..

[84] Wen Y.H., Lee T.Y., Fu P.C., Lo C.L., Chiang Y.T. (2017). Multifunctional Polymer Nanoparticles for Dual Drug Release and Cancer Cell Targeting. Polymers.

[85] Gao Y.-J., Qiao Z.-Y., Wang H. (2016). Polymers with Tertiary Amine Groups for Drug Delivery and Bioimaging. Sci. China Chem..

[86] Yang Q., Dong Y., Wang X., Lin Z., Yan M., Wang W., Dong A., Zhang J., Huang P., Wang C. (2021). pH-Sensitive Polycations for Sirna Delivery: Effect of Asymmetric Structures of Tertiary Amine Groups. Macromol. Biosci..

[87] Ueda H., Wakabayashi S., Kikuchi J., Ida Y., Kadota K., Tozuka Y. (2015). Anomalous Role Change of Tertiary Amino and Ester Groups as Hydrogen Acceptors in Eudragit E Based Solid Dispersion Depending on the Concentration of Naproxen. Mol. Pharm..

[88] Zhou L., Hou B., Wang D., Sun F., Song R., Shao Q., Wang H., Yu H., Li Y. (2020). Engineering Polymeric Prodrug Nanoplatform for Vaccination Immunotherapy of Cancer. Nano Lett..

[89] Banks S.R., Enck K., Wright M., Opara E.C., Welker M.E. (2019). Chemical Modification of Alginate for Controlled Oral Drug Delivery. J. Agric. Food Chem..

[90] Liu B., Thayumanavan S. (2017). Substituent Effects on the pH Sensitivity of Acetals and Ketals and Their Correlation with Encapsulation Stability in Polymeric Nanogels. J. Am. Chem. Soc..

[91] Zhai Y., Zhou X., Jia L., Ma C., Song R., Deng Y., Hu X., Sun W. (2017). Acetal-Linked Paclitaxel Polymeric Prodrug Based on Functionalized mPEG-PCL Diblock Polymer for pH-Triggered Drug Delivery. Polymers.

[92] Nuhn L., Vanparijs N., De Beuckelaer A., Lybaert L., Verstraete G., Deswarte K., Lienenklaus S., Shukla N.M., Salyer A.C., Lambrecht B.N. (2016). pH-Degradable Imidazoquinoline-Ligated Nanogels for Lymph Node-Focused Immune Activation. Proc. Natl. Acad. Sci. USA.

[93] Chen M., Xiang R., Wen Y., Xu G., Wang C., Luo S., Yin T., Wei X., Shao B., Liu N. (2015). A Whole-Cell Tumor Vaccine Modified to Express Fibroblast Activation Protein Induces Antitumor Immunity against Both Tumor Cells and Cancer-Associated Fibroblasts. Sci. Rep..

[94] Mollazadeh S., Mackiewicz M., Yazdimamaghani M. (2021). Recent Advances in the Redox-Responsive Drug Delivery Nanoplatforms: A Chemical Structure and Physical Property Perspective. Mater. Sci. Eng. C Mater. Biol. Appl..

[95] Doskey C.M., Buranasudja V., Wagner B.A., Wilkes J.G., Du J., Cullen J.J., Buettner G.R. (2016). Tumor Cells Have Decreased Ability to Metabolize H_2_O_2_: Implications for Pharmacological Ascorbate in Cancer Therapy. Redox Biol..

[96] Chen C., Tao R., Ding D., Kong D., Fan A., Wang Z., Zhao Y. (2017). Ratiometric Co-Delivery of Multiple Chemodrugs in a Single Nanocarrier. Eur. J. Pharm. Sci. Off. J. Eur. Fed. Pharm. Sci..

[97] Ramasamy T., Ruttala H.B., Chitrapriya N., Poudal B.K., Choi J.Y., Kim S.T., Youn Y.S., Ku S.K., Choi H.G., Yong C.S. (2017). Engineering of Cell Microenvironment-Responsive Polypeptide Nanovehicle Co-Encapsulating a Synergistic Combination of Small Molecules for Effective Chemotherapy in Solid Tumors. Acta Biomater..

[98] Fu S., Rempson C.M., Puche V., Zhao B., Zhang F. (2022). Construction of Disulfide Containing Redox-Responsive Polymeric Nanomedicine. Methods (San Diego Calif.).

[99] Xiao C., Ding J., Ma L., Yang C., Zhuang X., Chen X. (2014). Synthesis of Thermal and Oxidation Dual Responsive Polymer for Reactive Oxygen Species (ROS)-Triggered Drug Release. Polym. Chem..

[100] Mirhadi E., Mashreghi M., Faal Maleki M., Alavizadeh S.H., Arabi L., Badiee A., Jaafari M.R. (2020). Redox-Sensitive Nanoscale Drug Delivery Systems for Cancer Treatment. Int. J. Pharm..

[101] Manconi M., Manca M.L., Caddeo C., Cencetti C., di Meo C., Zoratto N., Nacher A., Fadda A.M., Matricardi P. (2018). Preparation of Gellan-Cholesterol Nanohydrogels Embedding Baicalin and Evaluation of Their Wound Healing Activity. Eur. J. Pharm. Biopharm..

[102] Gong C., Shan M., Li B., Wu G. (2016). A pH and Redox Dual Stimuli-Responsive Poly(Amino Acid) Derivative for Controlled Drug Release. Colloids Surf. B Biointerfaces.

[103] Wang H.Y., Wang R.F. (2012). Enhancing Cancer Immunotherapy by Intracellular Delivery of Cell-Penetrating Peptides and Stimulation of Pattern-Recognition Receptor Signaling. Adv. Immunol..

[104] Deng S., Iscaro A., Zambito G., Mijiti Y., Minicucci M., Essand M., Lowik C., Muthana M., Censi R., Mezzanotte L. (2021). Development of a New Hyaluronic Acid Based Redox-Responsive Nanohydrogel for the Encapsulation of Oncolytic Viruses for Cancer Immunotherapy. Nanomaterials.

[105] Chen Y., Xia R., Huang Y., Zhao W., Li J., Zhang X., Wang P., Venkataramanan R., Fan J., Xie W. (2016). An Immunostimulatory Dual-Functional Nanocarrier That Improves Cancer Immunochemotherapy. Nat. Commun..

[106] Sun J.J., Chen Y.C., Huang Y.X., Zhao W.C., Liu Y.H., Venkataramanan R., Lu B.F., Li S. (2017). Programmable Co-Delivery of the Immune Checkpoint Inhibitor NLG919 and Chemotherapeutic Doxorubicin Via a Redox-Responsive Immunostimulatory Polymeric Prodrug Carrier. Acta Pharmacol. Sin..

[107] Sun B., Luo C., Yu H., Zhang X., Chen Q., Yang W., Wang M., Kan Q., Zhang H., Wang Y. (2018). Disulfide Bond-Driven Oxidation- and Reduction-Responsive Prodrug Nanoassemblies for Cancer Therapy. Nano Lett..

[108] Sun B., Luo C., Zhang X., Guo M., Sun M., Yu H., Chen Q., Yang W., Wang M., Zuo S. (2019). Probing the Impact of Sulfur/Selenium/Carbon Linkages on Prodrug Nanoassemblies for Cancer Therapy. Nat. Commun..

[109] Li T., Pan S., Gao S., Xiang W., Sun C., Cao W., Xu H. (2020). Diselenide-Pemetrexed Assemblies for Combined Cancer Immuno-, Radio-, and Chemotherapies. Angew. Chem..

[110] Gao S., Li T., Guo Y., Sun C., Xianyu B., Xu H. (2020). Selenium-Containing Nanoparticles Combine the Nk Cells Mediated Immunotherapy with Radiotherapy and Chemotherapy. Adv. Mater..

[111] Bikram M., West J.L. (2008). Thermo-Responsive Systems for Controlled Drug Delivery. Expert Opin. Drug Deliv..

[112] Li J., Wang B., Liu P. (2008). Possibility of Active Targeting to Tumor by Local Hyperthermia with Temperature-Sensitive Nanoparticles. Med. Hypotheses.

[113] Bobbala S., Tamboli V., McDowell A., Mitra A.K., Hook S. (2016). Novel Injectable Pentablock Copolymer Based Thermoresponsive Hydrogels for Sustained Release Vaccines. AAPS J..

[114] Yang F., Shi K., Hao Y., Jia Y., Liu Q., Chen Y., Pan M., Yuan L., Yu Y., Qian Z. (2021). Cyclophosphamide Loaded Thermo-Responsive Hydrogel System Synergize with a Hydrogel Cancer Vaccine to Amplify Cancer Immunotherapy in a Prime-Boost Manner. Bioact. Mater..

[115] Tsai H.C., Chou H.Y., Chuang S.H., Lai J.Y., Chen Y.S., Wen Y.H., Yu L.Y., Lo C.L. (2019). Preparation of Immunotherapy Liposomal-Loaded Thermal-Responsive Hydrogel Carrier in the Local Treatment of Breast Cancer. Polymers.

[116] Wang F., Duan H., Xu W., Sheng G., Sun Z., Chu H. (2022). Light-Activated Nanomaterials for Tumor Immunotherapy. Front. Chem..

[117] Karimi M., Sahandi Zangabad P., Baghaee-Ravari S., Ghazadeh M., Mirshekari H., Hamblin M.R. (2017). Smart Nanostructures for Cargo Delivery: Uncaging and Activating by Light. J. Am. Chem. Soc..

[118] Maruoka Y., Furusawa A., Okada R., Inagaki F., Fujimura D., Wakiyama H., Kato T., Nagaya T., Choyke P.L., Kobayashi H. (2020). Combined CD44- and CD25-Targeted near-Infrared Photoimmunotherapy Selectively Kills Cancer and Regulatory T Cells in Syngeneic Mouse Cancer Models. Cancer Immunol. Res..

[119] Kobayashi H., Choyke P.L. (2019). Near-Infrared Photoimmunotherapy of Cancer. Acc. Chem. Res..

[120] Wei X., Song M., Jiang G., Liang M., Chen C., Yang Z., Zou L. (2022). Progress in Advanced Nanotherapeutics for Enhanced Photodynamic Immunotherapy of Tumor. Theranostics.

[121] Nakajima K., Miyazaki F., Terada K., Takakura H., Suzuki M., Ogawa M. (2021). Comparison of Low-Molecular-Weight Ligand and Whole Antibody in Prostate-Specific Membrane Antigen Targeted near-Infrared Photoimmunotherapy. Int. J. Pharm..

[122] Espinosa A., Di Corato R., Kolosnjaj-Tabi J., Flaud P., Pellegrino T., Wilhelm C. (2016). Duality of Iron Oxide Nanoparticles in Cancer Therapy: Amplification of Heating Efficiency by Magnetic Hyperthermia and Photothermal Bimodal Treatment. ACS Nano.

[123] Ou W., Jiang L., Thapa R.K., Soe Z.C., Poudel K., Chang J.H., Ku S.K., Choi H.G., Yong C.S., Kim J.O. (2018). Combination of NIR Therapy and Regulatory T Cell Modulation Using Layer-by-Layer Hybrid Nanoparticles for Effective Cancer Photoimmunotherapy. Theranostics.

[124] Zhang N., Song J., Liu Y., Liu M., Zhang L., Sheng D., Deng L., Yi H., Wu M., Zheng Y. (2019). Photothermal Therapy Mediated by Phase-Transformation Nanoparticles Facilitates Delivery of Anti-PD_1_ Antibody and Synergizes with Antitumor Immunotherapy for Melanoma. J. Control. Release.

[125] Bilal M., Iqbal H.M.N. (2019). Naturally-Derived Biopolymers: Potential Platforms for Enzyme Immobilization. Int. J. Biol. Macromol..

[126] Liu D., Nikoo M., Boran G., Zhou P., Regenstein J.M. (2015). Collagen and Gelatin. Annu. Rev. Food Sci. Technol..

[127] Caillol S. (2020). Special Issue “Natural Polymers and Biopolymers II”. Molecules.

[128] Castro J.G., Chin-Beckford N. (2015). Crofelemer for the Symptomatic Relief of Non-Infectious Diarrhea in Adult Patients with HIV/AIDS on Anti-Retroviral Therapy. Expert Rev. Clin. Pharmacol..

[129] Garcia-Martinez R., Caraceni P., Bernardi M., Gines P., Arroyo V., Jalan R. (2013). Albumin: Pathophysiologic Basis of Its Role in the Treatment of Cirrhosis and Its Complications. Hepatology.

[130] Rozga J., Piątek T., Małkowski P. (2013). Human Albumin: Old, New, and Emerging Applications. Ann. Transplant..

[131] Parodi A., Miao J., Soond S.M., Rudzińska M., Zamyatnin A.A. (2019). Albumin Nanovectors in Cancer Therapy and Imaging. Biomolecules.

[132] Tiwari R., Sethiya N.K., Gulbake A.S., Mehra N.K., Murty U.S.N., Gulbake A. (2021). A Review on Albumin as a Biomaterial for Ocular Drug Delivery. Int. J. Biol. Macromol..

[133] Wang R., Kim K.H., Yoo J., Li X., Kwon N., Jeon Y.H., Shin S.K., Han S.S., Lee D.S., Yoon J. (2022). A Nanostructured Phthalocyanine/Albumin Supramolecular Assembly for Fluorescence Turn-on Imaging and Photodynamic Immunotherapy. ACS Nano.

[134] Pham L.M., Poudel K., Ou W., Phung C.D., Nguyen H.T., Nguyen B.L., Karmacharya P., Pandit M., Chang J.H., Jeong J.H. (2021). Combination Chemotherapeutic and Immune-Therapeutic Anticancer Approach Via Anti-PD-L1 Antibody Conjugated Albumin Nanoparticles. Int. J. Pharm..

[135] Boran G., Regenstein J.M. (2010). Fish Gelatin. Adv. Food Nutr. Res..

[136] Djagny V.B., Wang Z., Xu S. (2001). Gelatin: A Valuable Protein for Food and Pharmaceutical Industries: Review. Crit. Rev. Food Sci. Nutr..

[137] Estevez J., Yang J.D., Leong J., Nguyen P., Giama N.H., Zhang N., Ali H.A., Lee M.H., Cheung R., Roberts L. (2019). Clinical Features Associated with Survival Outcome in African-American Patients with Hepatocellular Carcinoma. Am. J. Gastroenterol..

[138] Kim J., Francis D.M., Sestito L.F., Archer P.A., Manspeaker M.P., O’Melia M.J., Thomas S.N. (2022). Thermosensitive Hydrogel Releasing Nitric Oxide Donor and Anti-CTLA-4 Micelles for Anti-Tumor Immunotherapy. Nat. Commun..

[139] Bu L.L., Wang H.Q., Pan Y., Chen L., Wu H., Wu X., Zhao C., Rao L., Liu B., Sun Z.J. (2021). Gelatinase-Sensitive Nanoparticles Loaded with Photosensitizer and Stat3 Inhibitor for Cancer Photothermal Therapy and Immunotherapy. J. Nanobiotechnology.

[140] Xu X., Jha A.K., Harrington D.A., Farach-Carson M.C., Jia X. (2012). Hyaluronic Acid-Based Hydrogels: From a Natural Polysaccharide to Complex Networks. Soft Matter.

[141] Pereira H., Sousa D.A., Cunha A., Andrade R., Espregueira-Mendes J., Oliveira J.M., Reis R.L. (2018). Hyaluronic Acid. Adv. Exp. Med. Biol..

[142] Zhao Y., Zhang T., Duan S., Davies N.M., Forrest M.L. (2014). CD44-Tropic Polymeric Nanocarrier for Breast Cancer Targeted Rapamycin Chemotherapy. Nanomed. Nanotechnol. Biol. Med..

[143] Kim J.H., Moon M.J., Kim D.Y., Heo S.H., Jeong Y.Y. (2018). Hyaluronic Acid-Based Nanomaterials for Cancer Therapy. Polymers.

[144] Zhuang B., Chen T., Huang Y., Xiao Z., Jin Y. (2022). Chemo-Photothermal Immunotherapy for Eradication of Orthotopic Tumors and Inhibition of Metastasis by Intratumoral Injection of Polydopamine Versatile Hydrogels. Acta Pharm. Sinica B.

[145] Wang X., Lu J., Mao Y., Zhao Q., Chen C., Han J., Han M., Yuan H., Wang S. (2022). A Mutually Beneficial Macrophages-Mediated Delivery System Realizing Photo/Immune Therapy. J. Control. Release.

[146] Ali A., Ahmed S. (2018). A Review on Chitosan and Its Nanocomposites in Drug Delivery. Int. J. Biol. Macromol..

[147] Peluso G., Petillo O., Ranieri M., Santin M., Ambrosio L., Calabró D., Avallone B., Balsamo G. (1994). Chitosan-Mediated Stimulation of Macrophage Function. Biomaterials.

[148] Garg U., Chauhan S., Nagaich U., Jain N. (2019). Current Advances in Chitosan Nanoparticles Based Drug Delivery and Targeting. Adv. Pharm. Bull..

[149] Elieh-Ali-Komi D., Hamblin M.R. (2016). Chitin and Chitosan: Production and Application of Versatile Biomedical Nanomaterials. Int. J. Adv. Res..

[150] Alizadeh L., Zarebkohan A., Salehi R., Ajjoolabady A., Rahmati-Yamchi M. (2019). Chitosan-Based Nanotherapeutics for Ovarian Cancer Treatment. J. Drug Target..

[151] Mushtaq A., Li L., Anitha A., Grøndahl L. (2021). Chitosan Nanomedicine in Cancer Therapy: Targeted Delivery and Cellular Uptake. Macromol. Biosci..

[152] Yang X., Yu T., Zeng Y., Lian K., Zhou X., Ke J., Li Y., Yuan H., Hu F. (2020). pH-Responsive Biomimetic Polymeric Micelles as Lymph Node-Targeting Vaccines for Enhanced Antitumor Immune Responses. Biomacromolecules.

[153] Chen R., Xu J., Wu W., Wen Y., Lu S., El-Seedi H.R., Zhao C. (2022). Structure-Immunomodulatory Activity Relationships of Dietary Polysaccharides. Curr. Res. Food Sci..

[154] Geshi N., Petersen B.L., Scheller H.V. (2010). Toward Tailored Synthesis of Functional Polysaccharides in Plants. Ann. N. Y. Acad. Sci..

[155] Yin M., Zhang Y., Li H. (2019). Advances in Research on Immunoregulation of Macrophages by Plant Polysaccharides. Front. Immunol..

[156] Varghese S., Joseph M.M., Aravind S.R., Unnikrishnan B.S., Pillai K.R., Sreelekha T.T. (2019). Immunostimulatory Plant Polysaccharides Impede Cancer Progression and Metastasis by Avoiding Off-Target Effects. Int. Immunopharmacol..

[157] Jain A.K., Sahu H., Mishra K., Thareja S. (2021). Mannose Conjugated Starch Nanoparticles for Preferential Targeting of Liver Cancer. Curr. Drug Deliv..

[158] Yassaroh Y., Woortman A.J.J., Loos K. (2021). Physicochemical Properties of Heat-Moisture Treated, Stearic Acid Complexed Starch: The Effect of Complexation Time and Temperature. Int. J. Biol. Macromol..

[159] Lachowicz M., Stańczak A., Kołodziejczyk M. (2020). Characteristic of Cyclodextrins: Their Role and Use in the Pharmaceutical Technology. Curr. Drug Targets.

[160] Parrot-Lopez H., Perret F., Bertino-Ghera B. (2010). Amphiphilic Cyclodextrins and Their Applications. Preparation of Nanoparticles Based on Amphiphilic Cyclodextrins for Biomedical Applications. Ann. Pharm. Fr..

[161] Tian B., Hua S., Liu J. (2020). Cyclodextrin-Based Delivery Systems for Chemotherapeutic Anticancer Drugs: A Review. Carbohydr. Polym..

[162] Zhang Y., Ma S., Liu X., Xu Y., Zhao J., Si X., Li H., Huang Z., Wang Z., Tang Z. (2021). Supramolecular Assembled Programmable Nanomedicine as in Situ Cancer Vaccine for Cancer Immunotherapy. Adv. Mater..

[163] Paraman I., Lamsal B.P. (2011). Recovery and Characterization of A-Zein from Corn Fermentation Coproducts. J. Agric. Food Chem..

[164] Elzoghby A., Freag M., Mamdouh H., Elkhodairy K. (2017). Zein-Based Nanocarriers as Potential Natural Alternatives for Drug and Gene Delivery: Focus on Cancer Therapy. Curr. Pharm. Des..

[165] Tran P.H.L., Duan W., Lee B.J., Tran T.T.D. (2019). The Use of Zein in the Controlled Release of Poorly Water-Soluble Drugs. Int. J. Pharm..

[166] Lee S., Alwahab N.S., Moazzam Z.M. (2013). Zein-Based Oral Drug Delivery System Targeting Activated Macrophages. Int. J. Pharm..

[167] Dong F., Dong X., Zhou L., Xiao H., Ho P.Y., Wong M.S., Wang Y. (2016). Doxorubicin-Loaded Biodegradable Self-Assembly Zein Nanoparticle and Its Anti-Cancer Effect: Preparation, in Vitro Evaluation, and Cellular Uptake. Colloids Surf. B Biointerfaces.

[168] Soe Z.C., Ou W., Gautam M., Poudel K., Kim B.K., Pham L.M., Phung C.D., Jeong J.H., Jin S.G., Choi H.G. (2019). Development of Folate-Functionalized Pegylated Zein Nanoparticles for Ligand-Directed Delivery of Paclitaxel. Pharmaceutics.

[169] Qi Z., Pei P., Zhang Y., Chen H., Yang S., Liu T., Zhang Y., Yang K. (2022). ^131^I-αDP-L1 Immobilized by Bacterial Cellulose for Enhanced Radio-Immunotherapy of Cancer. J. Control. Release.

[170] Takahashi R.Y.U., Castilho N.A.S., Silva M., Miotto M.C., Lima A.O.S. (2017). Prospecting for Marine Bacteria for Polyhydroxyalkanoate Production on Low-Cost Substrates. Bioengineering.

[171] Iliou K., Kikionis S., Ioannou E., Roussis V. (2022). Marine Biopolymers as Bioactive Functional Ingredients of Electrospun Nanofibrous Scaffolds for Biomedical Applications. Mar. Drugs.

[172] Rahman M.A. (2021). Marine Skeletal Biopolymers and Proteins and Their Biomedical Application. Mar. Drugs.

[173] Fitton J.H., Stringer D.N., Karpiniec S.S. (2015). Therapies from Fucoidan: An Update. Mar. Drugs.

[174] Nishiya N., Oku Y., Ishikawa C., Fukuda T., Dan S., Mashima T., Ushijima M., Furukawa Y., Sasaki Y., Otsu K. (2021). Lamellarin 14, a Derivative of Marine Alkaloids, Inhibits the T790m/C797s Mutant Epidermal Growth Factor Receptor. Cancer Sci..

[175] Park H.B., Hwang J., Lim S.M., Zhang W., Jin J.O. (2020). Dendritic Cell-Mediated Cancer Immunotherapy with *Ecklonia cava* Fucoidan. Int. J. Biol. Macromol..

[176] Zhang W., Hwang J., Yadav D., An E.K., Kwak M., Lee P.C., Jin J.O. (2021). Enhancement of Immune Checkpoint Inhibitor-Mediated Anti-Cancer Immunity by Intranasal Treatment of *Ecklonia cava* Fucoidan against Metastatic Lung Cancer. Int. J. Mol. Sci..

[177] Chung C.H., Lu K.Y., Lee W.C., Hsu W.J., Lee W.F., Dai J.Z., Shueng P.W., Lin C.W., Mi F.L. (2020). Fucoidan-Based, Tumor-Activated Nanoplatform for Overcoming Hypoxia and Enhancing Photodynamic Therapy and Antitumor Immunity. Biomaterials.

[178] Su X., Cao Y., Liu Y., Ouyang B., Ning B., Wang Y., Guo H., Pang Z., Shen S. (2021). Localized Disruption of Redox Homeostasis Boosting Ferroptosis of Tumor by Hydrogel Delivery System. Mater. Today. Bio..

[179] Smith A.M., Senior J.J. (2021). Alginate Hydrogels with Tuneable Properties. Adv. Biochem. Eng. Biotechnol..

[180] Zhang C., Shi G., Zhang J., Song H., Niu J., Shi S., Huang P., Wang Y., Wang W., Li C. (2017). Targeted Antigen Delivery to Dendritic Cell Via Functionalized Alginate Nanoparticles for Cancer Immunotherapy. J. Control. Release.

[181] Pei M., Li H., Zhu Y., Lu J., Zhang C. (2022). In Vitro Evidence of Oncofetal Antigen and TLR-9 Agonist Co-Delivery by Alginate Nanovaccines for Liver Cancer Immunotherapy. Biomater. Sci..

[182] Aziz E., Batool R., Khan M.U., Rauf A., Akhtar W., Heydari M., Rehman S., Shahzad T., Malik A., Mosavat S.H. (2020). An Overview on Red Algae Bioactive Compounds and Their Pharmaceutical Applications. J. Complement. Integr. Med..

[183] Buck C.B., Thompson C.D., Roberts J.N., Müller M., Lowy D.R., Schiller J.T. (2006). Carrageenan Is a Potent Inhibitor of Papillomavirus Infection. PLoS Pathog..

[184] Zhang Y.Q., Tsai Y.C., Monie A., Hung C.F., Wu T.C. (2010). Carrageenan as an Adjuvant to Enhance Peptide-Based Vaccine Potency. Vaccine.

[185] Li J., Aipire A., Li J., Zhu H., Wang Y., Guo W., Li X., Yang J., Liu C. (2017). Λ-Carrageenan Improves the Antitumor Effect of Dendritic Cellbased Vaccine. Oncotarget.

[186] El-Deeb N.M., Ibrahim O.M., Mohamed M.A., Farag M.M.S., Farrag A.A., El-Aassar M.R. (2022). Alginate/κ-Carrageenan Oral Microcapsules Loaded with Agaricus bisporus Polysaccharides Mh751906 for Natural Killer Cells Mediated Colon Cancer Immunotherapy. Int. J. Biol. Macromol..

[187] Sanadgol N., Wackerlig J. (2020). Developments of Smart Drug-Delivery Systems Based on Magnetic Molecularly Imprinted Polymers for Targeted Cancer Therapy: A Short Review. Pharmaceutics.

[188] Shae D., Becker K.W., Christov P., Yun D.S., Lytton-Jean A.K.R., Sevimli S., Ascano M., Kelley M., Johnson D.B., Balko J.M. (2019). Endosomolytic Polymersomes Increase the Activity of Cyclic Dinucleotide STING Agonists to Enhance Cancer Immunotherapy. Nat. Nanotechnol..

[189] Wang L., Wang M., Zhou B., Zhou F., Murray C., Towner R.A., Smith N., Saunders D., Xie G., Chen W.R. (2019). Pegylated Reduced-Graphene Oxide Hybridized with Fe_3_O_4_ Nanoparticles for Cancer Photothermal-Immunotherapy. J. Mater. Chem. B.

[190] Urbina F., Morales-Pison S., Maldonado E. (2020). Enzymatic Protein Biopolymers as a Tool to Synthetize Eukaryotic Messenger Ribonucleic Acid (mRNA) with Uses in Vaccination, Immunotherapy and Nanotechnology. Polymers.

[191] Malikmammadov E., Tanir T.E., Kiziltay A., Hasirci V., Hasirci N. (2018). PCL and PCL-Based Materials in Biomedical Applicaions. J. Biomater. Sci. Polym. Ed..

[192] Tyliszczak B., Drabczyk A., Kudłacik-Kramarczyk S., Rudnicka K., Gatkowska J., Sobczak-Kupiec A., Jampilek J. (2019). In Vitro Biosafety of Pro-Ecological Chitosan-Based Hydrogels Modified with Natural Substances. J. Biomed. Mater. Res. Part A.

[193] Sia D., Jiao Y., Martinez-Quetglas I., Kuchuk O., Villacorta-Martin C., Castro de Moura M., Putra J., Camprecios G., Bassaganyas L., Akers N. (2017). Identification of an Immune-Specific Class of Hepatocellular Carcinoma, Based on Molecular Features. Gastroenterology.

[194] Xie Z., Shen J., Sun H., Li J., Wang X. (2021). Polymer-Based Hydrogels with Local Drug Release for Cancer Immunotherapy. Biomed. Pharmacother. Biomed. Pharmacother..

